# The complete mitochondrial genome of *Paragonimus ohirai* (Paragonimidae: Trematoda: Platyhelminthes) and its comparison with *P. westermani* congeners and other trematodes

**DOI:** 10.7717/peerj.7031

**Published:** 2019-06-20

**Authors:** Thanh Hoa Le, Khue Thi Nguyen, Nga Thi Bich Nguyen, Huong Thi Thanh Doan, Takeshi Agatsuma, David Blair

**Affiliations:** 1 Immunology Department, Institute of Biotechnology (IBT), Vietnam Academy of Science and Technology (VAST), Hanoi, Vietnam; 2 Graduate University of Science and Technology (GUST), Vietnam Academy of Science and Technology (VAST), Hanoi, Vietnam; 3 Department of Environmental Medicine, Kochi Medical School, Kochi University, Oko, Nankoku City, Kochi, Japan; 4 College of Science and Engineering, James Cook University, Townsville, Australia

**Keywords:** *Paragonimus ohirai*, *Paragonimus westermani*, Repeats, Skewness value, Phylogenetic analysis, Mitochondrial genome, Paragonimidae

## Abstract

We present the complete mitochondrial genome of *Paragonimus ohirai* Miyazaki, 1939 and compare its features with those of previously reported mitochondrial genomes of the pathogenic lung-fluke, *Paragonimus westermani*, and other members of the genus. The circular mitochondrial DNA molecule of the single fully sequenced individual of *P. ohirai* was 14,818 bp in length, containing 12 protein-coding, two ribosomal RNA and 22 transfer RNA genes. As is common among trematodes, an *atp8* gene was absent from the mitogenome of *P. ohirai* and the 5′ end of *nad4* overlapped with the 3′ end of *nad4L* by 40 bp*. Paragonimusohirai* and four forms/strains of *P. westermani* from South Korea and India, exhibited remarkably different base compositions and hence codon usage in protein-coding genes. In the fully sequenced *P. ohirai* individual, the non-coding region started with two long identical repeats (292 bp each), separated by *tRNA^Glu^*. These were followed by an array of six short tandem repeats (STR), 117 bp each. Numbers of the short tandem repeats varied among *P. ohirai* individuals. A phylogenetic tree inferred from concatenated mitochondrial protein sequences of 50 strains encompassing 42 species of trematodes belonging to 14 families identified a monophyletic Paragonimidae in the class Trematoda. Characterization of additional mitogenomes in the genus *Paragonimus* will be useful for biomedical studies and development of molecular tools and mitochondrial markers for diagnostic, identification, hybridization and phylogenetic/epidemiological/evolutionary studies.

## Introduction

Paragonimiasis, a neglected disease caused by lung flukes of the genus *Paragonimus* (Paragonimidae: Trematoda: Platyhelminthes), is a health threat in many tropical and subtropical regions. About seven species in the genus are common pathogens of humans, who usually acquire infection by eating undercooked or raw freshwater crabs or crayfish containing encysted metacercariae. Infection may also occur by eating raw meat of mammalian paratenic hosts (Yoshida et al., 2016).

The genus *Paragonimus* includes several species complexes. The best known is the *P. westermani* complex, members of which can infect people mainly in Japan, Korea, China and The Philippines. However, the geographical range of this complex encompasses much of eastern and southern Asia (Blair et al., 2016). The *P. ohirai* complex, which is phylogenetically distinct from the well-known *P. westermani* complex (Blair et al., 2016), also occurs in southern and eastern Asia. *Paragonimus ohirai* (Miyazaki, 1939) itself is primarily coastal in distribution, using fresh- and brackish-water crabs as intermediate hosts, and matures in a range of rodents and other small mammals. It is known from Japan, Taiwan, Korea and mainland China with very occasional human infections reported (Miyazaki, 1991; Habe & Agatsuma T. Hirai, 1985; Agatsuma & Habe, 1986; Agatsuma et al., 1992; Ryu et al., 2000; Sugiyama et al., 2004; Blair, 2008; Blair, 2014). No mitochondrial genome (mitogenome) is available yet for any member of the *P. ohirai* complex. To date, studies on *P. ohirai* have mainly focused on morphological, physiological, immunological and protein identification/characterization (Habe & Agatsuma T. Hirai, 1985; Agatsuma et al., 1992; Fujino et al., 1996; Ikeda, Oikawa & Nishiyama, 1996; Ikeda, 2002; Ikeda, 2006). There have been relatively few studies on molecular recognition/variation and discrimination between this species and other members of *Paragonimus* (Agatsuma & Habe, 1986; Van Herwerden, Blair & Agatsuma, 1999a; Van Herwerden, Blair & Agatsuma, 1999b; Ryu et al., 2000; Blair, Agatsuma & Watanobe, 1997; Blair, Xu & Agatsuma, 1999; Blair et al., 2016).

Genomic information from mitogenomes of animals can be utilized for taxonomic identification, phylogenetic and population studies (Boore, 1999; Hardman & Hardman, 2006; Bernt et al., 2013; Wey-Fabrizius et al., 2013; Solà et al., 2015). A typical trematode mitogenome is a circular DNA molecule consisting of 12 protein-coding, 22 transfer RNA (*tRNA*) and two ribosomal RNA genes. Gene order tends to be highly conserved within the Order Plagiorchiida (that contains most trematodes, including paragonimids), but variable features, mainly repeats, do occur in the long non-coding regions of these genomes (Wey-Fabrizius et al., 2013; Le, Blair & McManus, 2002; Le et al., 2016). Currently, about 50 complete or near-complete mitogenomes are available from trematodes (Table S1). Many important families are not yet represented at all (Wey-Fabrizius et al., 2013), while other families, such as the Paragonimidae, are represented by data for only a few species. GenBank lists four mitogenomes for *P. westermani* of different ploidies and/or geographical origins. Coding regions are complete for all of these, but not all include the full non-coding region. Two of these mitogenomes are from eastern Asia, both from South Korea, a diploid (GenBank: AF540958, incomplete non-coding region) and a triploid (AF219379). Another two *P. westermani* mitogenomes available in Genbank, KM280646 (incomplete non-coding region) and KX943544, are from Northeast India.

Here, we provide genomic information from the complete mitochondrial genome for *Paragonimus ohirai*, determining the gene content, arrangement and composition, to compare features of this genome with those of the four available mitogenomes from *P. westermani* and of other members of the genus. We also assess the value of mitogenomes for resolution of the relationships between the trematode species.

## Materials and Methods

### Source of samples

The *P. ohirai* samples used in this study were from batches of stored adult specimens, raised in rats experimentally infected with metacercariae collected from the crab hosts (Habe & Agatsuma T. Hirai, 1985). These batches of specimens have been used in many previous studies on morphology, isoenzyme profiles, molecular characterization of this species and molecular taxonomy of *Paragonimus* spp. (e.g., Habe & Agatsuma Hirai, 1985; Agatsuma & Habe, 1986; Agatsuma et al., 1992; Sugiyama et al., 2004; Miyazaki, 1991, Van Herwerden, Blair & Agatsuma, 1999a; Van Herwerden, Blair & Agatsuma, 1999b; Fujino et al., 1996). Metacercariae from crabs and adults obtained from infected rats were morphologically consistent with *P. ohirai* as described by Miyazaki (1991); Miyazaki (1939) and Sugiyama et al. (2004). In all cases, the metacercariae collected from the crab hosts were spherical with a thin outer (O) and a thicker inner (I) cyst wall (Habe & Agatsuma T. Hirai, 1985; Sugiyama et al., 2004). Adult worms were around 7 mm long and 3 mm wide. The ventral sucker was larger than the oral sucker; the ovary was smaller than the testes and intricately branched; cuticular spines formed distinct comb-like groups (Sugiyama et al., 2004). These morphological features permit discrimination of *P. ohirai* from all other *Paragonimus* species (Tomimura, Arakawa & Ono, 1957; Tada, Nagano & Sato, 1969; Habe & Agatsuma T. Hirai, 1985; Sugiyama et al., 2004).

Briefly, albino rats, *Rattus norvegicus*, were infected with metacercariae from natural crab hosts (*Sesarma dehaani,* recently renamed as *Chiromantes dehaani*) (Sugiyama et al., 2004). Crabs were collected at Kinosaki (Hyogo, 35°37′0″N; 134°49′0″E) and Nagoya (35°10′0″N, 136° 55′0″E) Prefectures in Japan. Five- to eight-week-old male rats were orally infected with metacercariae using a stomach tube. Rats were kept in separate cages and fed a commercial diet. Adult worms were collected two months post-infection. The infected rats were bled to death under ether anesthesia (Habe & Agatsuma T. Hirai, 1985; Agatsuma & Habe, 1986).

### Genomic DNA extraction

Following morphological identification, worms were preserved in 70% ethanol. Three adult worms derived from Kinosaki Prefecture and three from Nagoya Prefecture, were individually used for genomic extraction. The mitogenome of one specimen from Kinosaki was completely sequenced, remaining specimens were used to investigate variation in structure of the non-coding region.

Total genomic DNA was extracted from individual specimens using the GeneJET™ Genomic DNA Purification Kit (Thermo Fisher Scientific Inc., Waltham, MA, USA) according to the manufacturer’s instructions. Genomic DNA was eluted in 50 µL of the elution buffer provided in the kit and stored at −20 °C until use. The concentration of DNA was estimated using a GBC UV/visible 911A spectrophotometer (GBC Scientific Equipment Pty. Ltd., Braeside, Australia). A working concentration of 50 ng/µL was prepared and 1 µL of this was used as template for short PCRs and 2 µL for long PCRs, in a 50 µL reaction volume.

### Molecular identification of specimens

A molecular approach was also used to confirm the identity of our specimens as *P. ohirai*. Partial sequences of the *cox1* gene were obtained from PCR products amplified using the primer pairs JB3F/JB4.5R (Table 1). A tree was inferred from an alignment of 224*cox1* nucleotide sequences (309 bp) of 19 *Paragonimus* species available in GenBank and in previous publications. Phylogenetic reconstruction was performed using maximum-likelihood analysis (ML) with the Tamura-Nei model in the MEGA 7 package (Kumar, Stecher & Tamura, 2016). This model had the best Bayesian information criterion value, as determined using MEGA. Bootstrap support for each node was evaluated using 100 bootstrap resamplings. The tree inferred showed all *P. ohirai* sequences together in one group with those of the closely related Japanese *P. iloktsuenensis* (Fig. S1). *Paragonimus iloktsuenensis* and *P. sadoensis* have been regarded as synonyms of *P. ohirai* (Blair, Agatsuma & Watanobe, 1997).

**Table 1:** Primers for amplification and sequencing of the mitochondrial genome of *Paragonimus ohirai*.

**Primer**	**Sequence (5′->3′)**	**Location**
URNLF^a^	AGCCAGGTTGGTTCTTATCTAT	*rrnL*
URNSR^a^	TACCWTGTTACGACTTAHCWCA	*rrnS*
TRECOBF^a^	CAGATGTCYTATTGGGCTGC	*cob*
TRECOBR^a^	GAACHRVCCACARYCCCTTAAAC	*cob*
JB3F^a^	TTTTTTGGGCATCCTGAGGTTTAT	*cox1*
JB4.5R^a^	TAAAGAAAGAACATAATGAAAATG	*cox1*
PO1R	ACATAGCCACTAACACAGCAC	*nad2*
PO2F	TGGTTCGGGTGTTTTCTGCG	*cob*
PO3R	CCCACTCAAAACGAAACCTAAG	*atp6*
PO4F	CGGTGAGGGTTCCTCTGTTG	*tRNA*^*Ala*^
PO5F	GTCTCACAATCCCAAATCCTGG	*cox1*
PO6F	GTTTGGGAAGTGTTGTCTGGG	*cox1*
PO7F	TCCTTTTAGGGGAGTAAAGGCC	*cob*
PO8R	GCACAACCGCAACAAAGACC	*atp6*
PO9R	AGCAGCCCAATCAGAGTTACC	*rrnS*
PO10F	CTGCACCAAATCCGAACGCC	*nad3*
PO11F	CTTATGGAACTGCAGCACTTC	*cox2*
PO12R	CGTACCCAACAAACATAAACC	*nad5*
PO13F	AATTTTTCGTGTTAGAGGGAG	*tRNA*^*Glu*^
PO14R	GAAGATAACGAATTAGCCAAAC	*cob*
PO15F	GTGGGTACTTGTATTCTGTG	*nad6*
PO16R	GTCAAAACACAAAACAAACAC	*nad5*
PO17R	CGCCGTAATTGAAGAACC	*cox3*
PO18F	GGTGTAGAAGGGTTGCAC	*tRNA*^*Glu*^
PO19F	GGCACTTTTTAAGGCTGGTGG	*nad5*
PO20R	ACCTCAGATTGGAAGGAAAGCAC	NR
PHOR	GTTACCAAAGGATCCGCCTGC	*cob*
PHOWR	CGGTATGTACCCCAACTAAATC	*rrnS*
POHF	ATGATTTGCAGGAGATTTCGGAC	*rrnS*
PHOGLUR	CCTCTAACACGAAAAATTAAC	*tRNA*^*Glu*^
GLYF^a^	AGTATKYYGTCTTTCCAAGTC	*tRNA*^*Gly*^
GLYR^a^	ACKAGACCHCYGACTTGGAAAGAC	*tRNA*^*Gly*^

**Notes.**

a Platyhelminth-universal primers used for inital amplification of the corresponding genes/regions to get sequences to design further primers.

### Amplification of the mitochondrial genome

The complete mitogenome was sequenced from one specimen from Kinosaki Prefecture. Initially, platyhelminth-universal primers (URNLF/URNSR for the *16S rRNA–12S rRNA*, TRECOBF/TRECOBR for *cytochrome b (cob)* and JB3F/JB4.5R for the *cox1* region), were used for amplification and subsequent sequencing of the corresponding genes/regions (Le, Blair & McManus, 2002; Le et al., 2016). The sequence data obtained were used to design further specific *P. ohirai* primers (Table 1).

Some specific primers in *nad5* or *cob* regions were used with the GLYF or GLYR platyhelminth-universal primers in long-PCRs. Long PCR was used to obtain medium-length and long fragments (5–8 kb) of overlapping regionsand conventional PCRs were used to amplify the rest of the mitogenome. All long-PCRs were prepared using AccuPower^®^ ProFi Taq PCR PreMixKit (Bioneer, Daejeon, South Korea) and short PCRs using Thermo Scientific DreamTaq PCR Master Kit (Thermo Fisher Scientific Inc., Waltham, MA, USA). All PCRs of 50 µL volume were performed in a MJ PTC-100 thermal cycler with initial denaturation at 95 °C for 5 min, followed by 35 cycles, each consisting of denaturation step for 30 s at 94 °C, annealing at 56 °C for 30 s, extension at 68 °C (in some cases, at 72 °C) for 3 to 10 min, depending on the lengths of the expected amplicons; and a final extension at 68 °C (or 72 °C for some reactions) for 7 or 10 min. A negative (no-DNA) control was included in some cases. The PCR products (5–10 µL of each) were examined on a 1% agarose gel, stained with ethidium bromide, and visualized under UV light (Wealtec, Sparks, NV, USA).

### Sequencing, assembling and annotation of the mitogenome

PCR products were purified using a GeneJET Gel Extraction and DNA Cleanup Micro Kit (Thermo Scientific Inc.). In some cases, PCR products were cloned using a CloneJET PCR Cloning Kit (if <2 kb) or a TOPO^®^ XL PCR Cloning Kit (Invitrogen Inc., Carlsbad, CA, USA) (if >2 kb). Plasmid DNA was extracted using a GeneJET Plasmid Miniprep Kit (Thermo Scientific, Inc.). A primer-walking approach to sequencing was used. PCR fragments and/or recombinant plasmid DNA were sequenced on automated sequencers using specific or M13 universal sequencing primers, respectively (Macrogen Inc., Seoul, South Korea). Both strands were completely sequenced and at least six sequences (three from each strand) were aligned to obtain the final sequence for characterization.

To explore size variation in the non-coding region (NR), we used forward primer GLYF (binding in *tRNA*^*Gly*^) and reverse primer PO17R (located in the *cox3* gene) (Table 1), spanning the full NR, for amplification and sequencing of this region from three additional *P. ohirai* individuals each from Kinosaki and from Nagoya.

Raw and edited sequences (edited using SeqEd v.1.3) were assembled and aligned using AssemblyLIGNv 1.9c and analyzed using the MacVector 8.2 package (Accelrys Inc.). Preliminary identity of a sequence or a region was assigned by searching the GenBank database (https://blast.ncbi.nlm.nih.gov/) using Basic-BLAST or two-sequence-BLAST, or by comparison with the corresponding platyhelminth sequences earlier obtained by us (Le, Blair & McManus, 2002; Le et al., 2016). Repeat sequences were detected in the long non-coding region (LNR) using the Tandem Repeats Finder v3.01 (Benson, 1999).

To confirm the length and to determine the composition and codon usage of each gene, comparisons of nucleotide and amino acid sequences of individual genes were done using MacVector 8.2 (Accelrys Inc.). The echinoderm/flatworm mitochondrial genetic code (translation table 9) was used in all programs for sequence analysis of mitogenomes of trematodes, including *P. ohirai*. Protein-coding genes were identified by the sequence similarities of translated amino acid sequences from their open reading frames to those already available in the GenBank database. Initiation codons other than ATG (specifically GTG) were considered. Codon usage and nucleotide composition were analyzed using MEGA 7 (Kumar, Stecher & Tamura, 2016). Codon usage for all the protein-coding genes was determined with the online program GENE INFINITY (Codon Usage: http://www.geneinfinity.org/sms/sms_codonusage.html). TreePuzzle v5.3 (Schmidt et al., 2002) was used to confirm (chi-square tests) that the amino-acid composition of each species did not significantly different from the average over the entire alignment (thus potentially biasing phylogenetic inference).

The transfer RNA genes (tRNA were identified using online software, primarily tRNAscan-SE v1.21 (Lowe & Eddy, 1997) with parameters specified for mitochondrial/chloroplast DNA. Three other separate software packages were also used online; ARWEN v 1.2 (Laslett & Canback, 2008); DOGMA (Wyman, Jansen & Boore, 2004) and MITOS Alpha version (Bernt et al., 2013). Final sequences and secondary structures were based on comparisons using all these programs. All transfer RNAs proposed by these programs were checked to confirm their typical “clover-leaf” structure or known variants of this. Any tRNAs not detected by these programs were found by inspection of the sequences, based on the alignment with other trematode tRNA sequences, to form tRNA structures.

Skew values (ranging from −1 to +1) were according to the formula defined by *Perna & Kocher (1995)* as: AT skew = (A − T)/(A + T) and GC skew = (G − C)/(G + C), where the letters stand for the usage of the corresponding nucleotides in the sequences (see also Solà et al., 2015).

### Phylogenetic reconstruction

Concatenated aligned sequences of 12 mitochondrial protein-coding genes from 50 strains/species of 14 families of trematodes listed in Table S1 were used in a phylogenetic analysis. In addition to *P. ohirai* (Kinosaki strain, Japan), the four available *P. westermani* sequences (two from South Korea and two from India) were included. Also included were two mitogenomes of *P. heterotremus* (accession numbers MH059809 and KY952166) and one of *P. kellicotti* (MH322000). Protein-coding nucleotide sequences were conceptually translated and the amino acid sequences for each gene were aligned separately using MAFFT (available at https://www.ebi.ac.uk/Tools/msa/mafft/) (Katoh & Standley, 2013). Subsequently, the alignments of all 12 genes were concatenated (final alignment length 3,660 amino acids) for phylogenetic analysis. The inferred amino acid sequences of individual species ranged in length from 3,314 amino acids (*Orientobilharzia turkestanicum* (GenBank: HQ283100; Schistosomatidae)) to 3,405 amino acids (*Metagonimus yokogawai* (GenBank: KC330755; Heterophyidae)). Most sequences ranged from 3,351 to 3,361 amino acids. Prior to phylogenetic analysis, regions of questionable alignment quality were removed using GBlocks (Castresana, 2000). This reduced the number of positions to 2,509 amino-acid residues.

Phylogenetic reconstruction was performed in MrBayes 3.2 program (Ronquist et al., 2012). The amino-acid model prior was set to “mixed”. This setting allows the program to infer the best fitting (from ten options) amino-acid substitution model. Two runs, each of four chains, were run for 1,000,000 generations, after which the standard deviation of split frequencies across runs was <0.01. Trees were sampled every 500 generations, and the first 25% discarded as burnin. The Jones, Taylor and Thornton model (Jones, Taylor & Thornton, 1992) was the only amino-acid model supported in the analysis (posterior probability 1.000 and standard deviation 0.000). The tree produced was a 50% majority-rule tree to which all compatible groupings were added. The tree was rooted by outgroup (i.e., members of the Schistosomatidae), a family known to be near the base of the trematode phylogeny in Olson et al. (2003).

**Figure 1:**
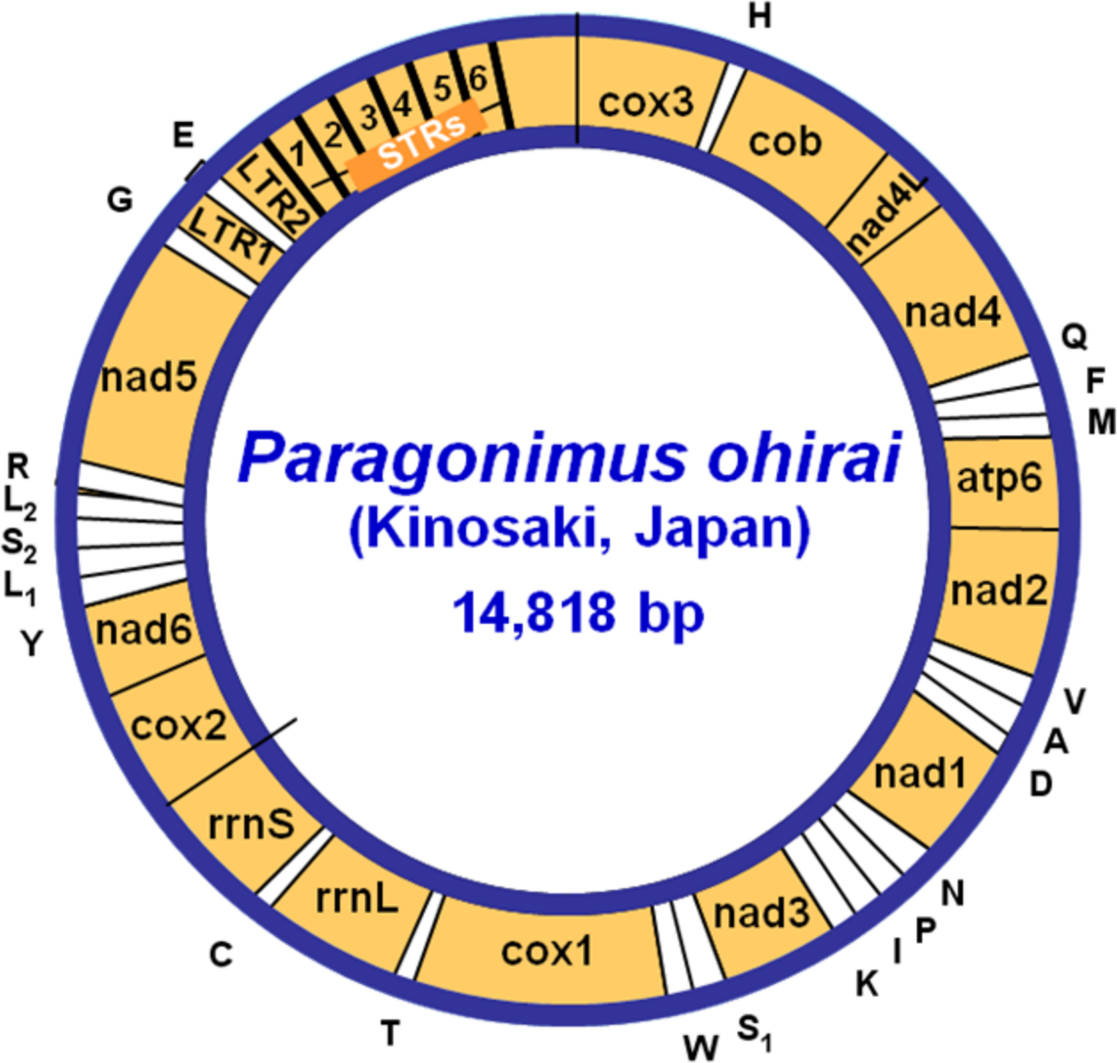
A circular map of the mitochondrial genome of the lung fluke *Paragonimus ohirai* (KX765277). Protein-coding and ribosomal large and small subunit genes are abbreviated according to *Boore (1999)* with slight modification by Le, Blair & McManus (2002). Genes are transcribed in a clockwise direction from one strand. The transfer RNA genes (*tRNA*) are designated by the single-letter code for the corresponding amino acid abbreviations, with the exception of those coding for Serine (*tRNA*^*Ser*1(*AGN*)^ and *tRNA*^*Ser*2(*UCN*)^) (Table 2). The long non-coding region consists of two long repeats (LR1 and LR2) separated by *tRNA*^*Glu*^, and followed by six short tandem repeats (STR). A unique sequence is between the last STR6 and *cox3*.

## Results and Discussion

### Gene organization, content and genomic features

The entire mitochondrial genome sequence obtained from the single fully sequenced *P. ohirai* adult (designated as Pohi-Kinosaki-JP) was deposited in GenBank with the accession number KX765277. This mtDNA was a circular molecule of 14,818 bp (Fig. 1). Locations of genes and other features in this genome are given in Table 2. This is within the length range commonly reported in trematodes, including *P. westermani* (Paragonimidae), *Echinochasmus japonicus* (Echinochasmidae), *Haplorchis taichui* (Heterophyidae), *Homalogaster paloniae* (Gastrodiscidae), *Dicrocoelium* spp. (Dicrocoeliidae) (Le, Blair & McManus, 2002; Le et al., 2016; Lee et al., 2013; Liu et al., 2014a; Brabec et al., 2015; Ma et al., 2016a; Yang et al., 2016a), but slightly shorter than in some schistosomes such as *Schistosoma spindale* (Schistosomatidae) and longer than in opisthorchiids, fasciolids and paramphistomids such as *Clonorchis sinensis, Opisthorchis viverrini, Fasciola hepatica, F. gigantica* and *Paramphistomum* spp. (Paramphistomidae) (Le, Blair & McManus, 2001a; Littlewood et al., 2006; Shekhovtsov et al., 2010; Cai et al., 2012; Liu et al., 2014b; Ma et al., 2015; Na et al., 2016). The mitochondrial genome of *P. ohirai* contained 36 genes: 12 protein-encoding genes, two ribosomal RNA genes and 22 transfer RNA genes (Table 2; Fig. 1). Genes abutted one another or were separated by a short intergenic sequence (Table 2; Fig. S2). The genomic organization of the coding portion in *P. ohirai* was identical with that in four available *P. westermani* mitogenomes and those of *P. heterotremus* and *P. kellicotti*, and similar to that of other trematodes including *Clonorchis sinensis* (Shekhovtsov et al., 2010), Opisthorchis viverrini (Cai et al., 2012), *Fasciola hepatica* (Le, Blair & McManus, 2001a), *Fasciola gigantica* (Liu et al., 2014b) and *Paramphistomum leydeni* (Ma et al., 2015), but different from some species of Schistosomatidae (Littlewood et al., 2006; Le et al., 2001b; ; Webster et al., 2007). An extensive non-coding region (NR) was found between *tRNA*^*Gly*^ and *cox3*. The NR was divided into two subregions, separated by *tRNA*^*Glu*^.

### Characteristics of protein-coding genes in *Paragonimus* species

In the *P. ohirai* mitogenome, ten protein-coding genes used ATG as the start codon and TAG as the stop codon and the remaining two genes were initiated with GTG and terminated with TAG and TAA codons (Table 2). *Cox1* and *cox2* genes were terminated by an incomplete TA- codon, which was presumably completed by the adjacent nucleotide, G (at the start of *tRNA*^*Thr*^ and*nad6*, respectively). Translations in all the remaining protein-coding genes were consistent with translation Table 9. Unusual initiation codons, such as ATA or ATT, reported from some metazoans, and TTG or GTT, reported from nematodes, were not found to initiate genes in *P. ohirai* (Table 2).

Base composition in protein-coding genes is very different between *P. ohirai* and the four mitogenomes of *P. westermani* (Table 3). In the former species, frequency of A is 17.00% (13.33–14.89% in *P. westermani*) and of T is 46.00% (38.21–40.50% in *P. westermani*). For both these bases, but particularly T, the base composition in *P. ohirai* is far closer to that typical of the other trematodes we included than that of *P. westermani* (Le, Blair & McManus, 2004) (Table 3). The G+C value in *P. ohirai* is lower than in the available *P. westermani* mitogenomes (Table 3), but closer to that in other trematodes (Le, Blair & McManus, 2004). In all the *Paragonimus* mitogenomes (as in trematodes generally), there is a strong AT skew: (−0.46 to −0.48). In *P. ohirai*, the preponderance of G over C is more marked (GC-skew 0.41) than in *P. westermani* (GC skew: 0.26 to 0.32) (Table 3), and closer to that seen in parasitic flatworms overall (Le, Blair & McManus, 2004). Base composition statistics for *P. heterotremus* and *P. kellicotti*, were intermediate between those for *P. ohirai* and *P. westermani*. All 64 codons in the mitochondrial genetic code table were used in the mtDNA protein-coding genes in *P. ohirai* (Table S1). There are 3,356 codons (excluding stop codons) in mitogenomes of *P. ohirai* and Indian *P. westermani* Type I (GenBank: KM280646), 3,355 codons in both Korean diploid and triploid *P. westermani* from East Asia and *P. heterotremus* and 3,354 in *P. kellicotti*. The remaining Indian *P. westermani* (from Arunachal Pradesh, KX943544) uses 3,368 codons (Table S1). An insertion of 45 nucleotides (15 amino acids) relative to other *Paragonimus* mitogenomes (and indeed all other trematodes) was found in the *cytochrome b* gene of this mitogenome. It remains to be determined whether this is a true feature unique to this Indian strain or whether it is due to mis-assembly. This region was omitted from phylogenetic analyses.

**Table 2:** Locations of genes and other features in the mitochondrial genome of *Paragonimus ohirai* (14,818 bp).

**Gene**	**Position(5′>3′)**	**Length**		**Codon**		**tRNAanti-codon**	**Identification of tRNA by**	**Int. seq.length (bp)**
		**bp**	**aa**	**start**	**stop**			
*cox3*	1–645	645	214	ATG	TAG			+3
*tRNA*^*His*^	649–717	69				GTG	AR, SE, DO	0
*cob*	718–1,836	1,119	372	ATG	TAG			+5
*nad4L*	1,842–2,105	264	87	ATG	TAG			−40
*nad4*	2,066–3,325	1,260	419	ATG	TAG			+11
*tRNA*^*Gln*^	3,337–3,398	62				TTG	AR	+6
*tRNA*^*Phe*^	3,405–3,470	66				GAA	AR, SE, DO	0
*tRNA*^*Met*^	3,471–3,535	65				CAT	AR, SE, DO	0
*atp6*	3,536–4,051	516	171	ATG	TAG			+2
*nad2*	4,054–4,920	867	288	ATG	TAG			+7
*tRNA*^*Val*^	4,928–4,991	64				TAC	AR, SE, DO	+2
*tRNA*^*Ala*^	4,994–5,059	66				TGC	AR, SE, DO	+6
*tRNA*^*Asp*^	5,066–5,132	67				GTC	AR, SE, DO	0
*nad1*	5,133–6,038	906	301	ATG	TAG			+11
*tRNA*^*Asn*^	6,049–6,119	70				GTT	AR, SE, DO	−2
*tRNA*^*Pro*^	6,118–6,184	67				TGG		+7
*ttRNA*^*Ile*^	6,192–6,254	63				GAT		+11
*tRNA*^*Lys*^	6,266–6,329	64				CTT	AR, SE, DO	0
*nad3*	6,330–6,686	357	118	ATG	TAG			+7
*tRNA*^*Ser*1(*AGN*)^^a^	6,694–6,755	62				GCT	AR	+7
*tRNA*^*Trp*^	6,763–6,827	65				TCA	AR, SE, DO	+3
*cox1*	6,831–8,363	1,533	510	ATG	TAG			−1
*tRNA*^*Thr*^	8,363–8,428	66				TGT	AR, SE, DO	0
*16S RNA*	8,429–9,402	974						0
*tRNA*^*Cys*^	9,403–9,468	66				GCA	AR, DO	0
*12S RNA*	9,469–10,204	736						0
*cox2*	10,205–10,804	600	199	ATG	TAG			−1
*nad6*	10,804–11,256	453	150	GTG	TAG			+3
*tRNA*^*Tyr*^	11,260–1,1,320	61				GTA	AR, DO	+11
*tRNA*^*Leu*1(*CUN*)^	11,332–11,396	65				TAG	AR, DO	−3
*tRNA*^*Ser*1(*UCN*)^^a^	11,394–11,457	64				TGA	AR	+18
*tRNA*^*Leu*2(*UUR*)^	11,476–11,538	63				TAA	AR, SE, DO	+11
*tRNA*^*Arg*^	11,540–11,618	69				TCG	AR, DO	0
*nad5*	11,619–13,202	1,584	527	GTG	TAA			+10
*tRNA*^*Gly*^	13,213–13,284	72				TCC	AR, SE, DO	+28
LR1	13,313–13,604	292						+8
*tRNA*^*Glu*^	13,613–13,681	69				TTC	SE, DO	−29
LR2	13,653–13,944	292						+15
STR1	13,960–14,076	117						0
STR2	14,077–14,193	117						0
STR3	14,194–14,310	117						0
STR4	14,311–14,426	117						0
STR5	14,427–14,544	117						0
STR6	14,545–14,661	117						0
unique seq	14,662–14,818	157						0

**Notes.**

bp basepair aa amino acid Int. seq. intergenic sequence

(+, number of nucleotides before start of the following gene; −, number of nucleotides overlapping with the following gene or other feature); LR, Long repeat; STR, short tandem repeat. Transfer RNAs (tRNA) found by software used in this study (SE] tRNAscan-SE 1.21 (Lowe & Eddy, 1997) AR, ARWEN Alpha version (Laslett & Canback, 2008); DO, DOGMA (Wyman, Jansen & Boore, 2004) with slight amendment own by our own determination.

a Indicating tRNAs lacking DHU-arm.

Codon usage indicated that codons containing T and/or A are far more common in *P. ohirai* and those containing C and/or G are less frequent than in *P. westermani* (Table S1). For example, for phenylalanine (Phe), the TTT codon is much more frequently used in *P. ohirai* (325 codons/9.65%) than in diploid and triploid *P. westermani* (208/6.18–6.19%), *P. westermani* type I (255/7.57%) and *P. westermani* AP (253/7.49%). Similar differences in frequencies are noted for the ATT codon for isoleucine (Ile), TTA for leucine (Leu), TCT for serine (Ser), and TAT for tyrosine (Tyr) (Table S1). These differences in base composition greatly influence the skew value calculation (Table 3).

### Ribosomal and transfer RNA genes

The mitochondrial large and small ribosomal subunit RNA genes (*16S rRNA* and *12S rRNA*, respectively) were located between the *tRNA*^*Thr*^ andthe *cox2* genes and were separated from each other by *tRNA*^*Cys*^, as in all platyhelminths reported to date (Le, Blair & McManus, 2002; Le et al., 2016; Liu et al., 2014a; Ma et al., 2016a; Ma et al., 2016b); 2017; (Shekhovtsov et al., 2010; Na et al., 2016; Biswal et al., 2014; Chen et al., 2016). The lengths of *16S rRNA* and *12S rRNA*, in the mitogenome of *P. ohirai,* were 974 bp and 736 bp, respectively (Table 2). As in the protein-coding genes, usage of T was high relative to A and usage of C was low relative to G in ribosomal genes. Similarly, the AT-skew was comparable for this region of all *Paragonimus* mitogenomes (−0.23 to −0.26) but the GC-skew was slightly higher in *P. ohirai* (0.35) than in *P. westermani* (0.29–0.34) (Table 3).

**Table 3:** **Base composition and related statistics (skewness evaluation) for protein-coding genes and ribosomal regions of the mtDNA of*Paragonimus ohirai*and other*Paragonimus*species, and means and standard deviations of base composition of all species included in the study**.

**Sequence**		Length(nt)*	**A**	**T**	**G**	**C**	**A+T**	**AT-skew**	**G+C**	**GC-skew**
Protein-coding	Pohi-Kinosaki-JP	10,104	**17.00**	**46.00**	26.17	10.83	63.00	−0.46	37.00	0.41
	Pwes(2n)-Haenam-KR	10,101	13.40	38.21	30.61	17.77	51.61	−0.48	48.39	0.26
	Pwes(3n)-Bogil-KR	10,101	13.33	38.25	30.61	17.81	51.58	−0.48	48.42	0.26
	Pwes-TypeI-IN	10,104	14.89	40.50	29.36	15.25	55.39	−0.46	44.61	0.32
	Pwes-AP-IN	10,140	14.72	40.40	29.58	15.30	55.12	−0.46	44.88	0.32
	Phet_China	10,101	14.93	43.91	28.18	12.98	58.84	−0.49	41.16	0.37
	Pkell	10,098	15.19	44.78	27.73	12.30	59.97	−0.49	40.30	0.38
Mean, all included species			17.58	46.04	25.91	10.41	63.64	−0.45	36.38	0.43
S.D. all included species			3.5	2.86	2.85	2.49	4.93	0.07	4.91	0.07
Ribosomal RNA coding	Pohi-Kinosaki-JP	1,710	22.92	37.19	26.90	12.98	60.01	−0.23	39.99	0.35
	Pwes(2n)-Haenam-KR	1,732	19.11	32.62	31.12	17.15	51.73	−0.26	48.27	0.29
	Pwes(3n)-Bogil-KR	1,732	18.94	32.79	31.29	16.97	51.73	−0.27	48.27	0.30
	Pwes-TypeI-IN	1,729	20.24	35.11	29.84	14.81	55.35	−0.27	44.65	0.34
	Pwes-AP-IN	1,721	20.22	34.63	30.16	14.99	54.85	−0.26	45.15	0.34
	Phet China	1,711	20.81	37.05	28.23	13.91	57.86	−0.28	42.14	0.34
	Pkell	1,711	21.33	37.52	27.47	13.68	58.85	−0.28	41.15	0.33

**Notes.**

Pohi *Paragonimus ohirai* Pwes *P. westermani* Phet *P. heterotremus* Pkell *P. kellicotti.* JP Japan KR South Korea IN India. Kinosaki Kinosaki locality, Japan AP Arunachal Pradesh locality, India (see Table S1)

High A and T nucleotide use (17.00% and 46.00%) in *P. ohirai* is bolded. S.D. standard deviation nt nucleotide.

a The overlap between *nad4L* and *nad4* is counted twice in this calculation.

**Figure 2:**
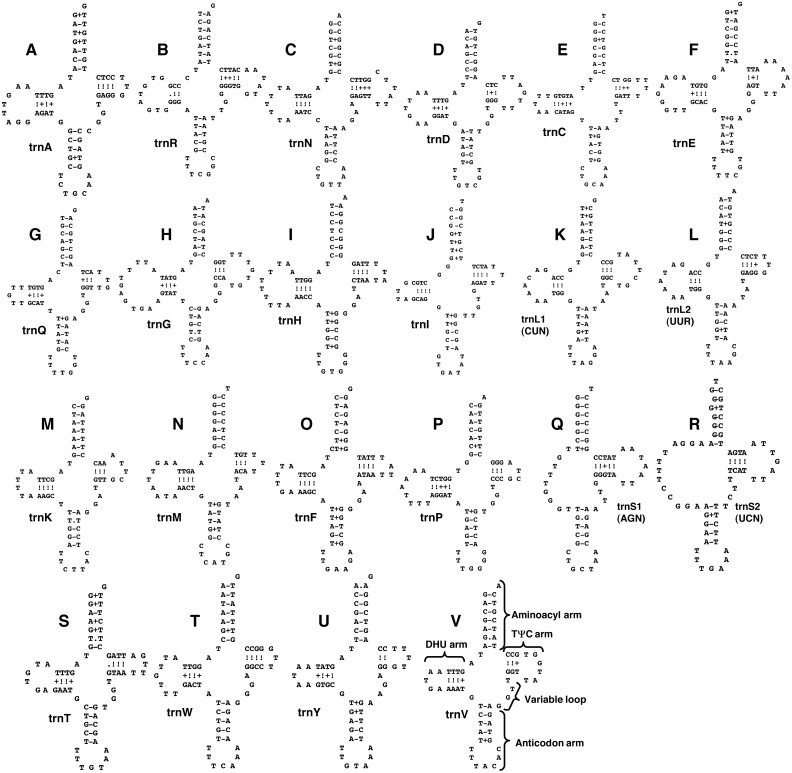
Drawings of predicted structure models of 22 transfer RNAs in the mitochondrial genome of *Paragonimus ohirai*, arranged in alphabetical order of the amino acids they specify. Each tRNA (here abbreviated as *trn*) gene is named according to the one-letter amino acid abbreviation, with the exception of those specifying Serine, S1 and S2; and Leucine, L1 and L2 (L1, CUN; L2, UUR; S1, AGN; and S2, UCN); DHU arms are missing in *tRNA*^*Ser*1(*AGN*)^ and in *tRNA*^*Ser*2(*UCN*)^. A: trnA (Alanine); B: trnR (Arginine); C: trnN (Asparagine); D: trnD (Aspartic acid); E: trnC (Cystine); F: trnE (Glutamic acid); G: trnQ (Glutamine); H: trnG (Glycine); I: trnH (Histidine); J: trnI (Isoleucine); K: trnL1(CUN) (Leucine); L: trnL2(UUR) (Leucine); M: trnK (Lysine); N: trnM (Methionine); O: trnF (Phenylalanine); P: trnP (Proline); Q: trnS1(AGN) (Serine); R: trnS2(UCN) (Serine); S: trnT (Threonine); T: trnW (Tryptophan); U: trnY (Tyrosine); V: trnV (Valine). Names of structural components of a tRNA gene are indicated in the *tRNA*^*Val*^ structure.

Twenty-two transfer RNAs were identified, ranging in size from 61 nucleotides (for *tRNA*^*Tyr*^) to 72 (for *tRNA*^*Gly*^): most are within the range 64–67 nucleotides (Table 2). All anticodon usage is consistent with those described for other platyhelminth species. Twenty of the tRNAs can be folded into typical ‘cloverleaf’ secondary structures. The remaining two tRNAs for serine, *tRNA*^*Ser*1(*AGN*)^ (62 bp) and *tRNA*^*Ser*2(*UCN*)^ (64 bp), contain the full T ΨC arm but not the dihydrouridine (DHU) arm (instead, it is replaced by a loop of ten nucleotides for the former and 13 for the latter) (Fig. 2). A DHU arm-lacking form for the former transfer RNA is usual in metazoans and is usual for the latter tRNA in platyhelminths and occurs in some other animal taxa, especially nematodes (Boore, 1999; Le, Blair & McManus, 2001a; Le, Blair & McManus, 2002; Le et al., 2016; Lee et al., 2013; Brabec et al., 2015; Ma et al., 2016a; Yang et al., 2016a; Littlewood et al., 2006; Wolstenholme, 1992). In *P. ohirai*, the *tRNA*^*Thr*^ gene uses the last nucleotide G of the protein-coding *cox1* gene termination codon; *tRNA*^*Pro*^ overlaps *tRNA*^*Asn*^ by 2 nucleotides; and *tRNA*^*Leu*1(*CUN*)^ overlaps *tRNA*^*Ser*2(*UCN*)^by 3 nucleotides in their sequences (Table 2). *tRNA*^*Gly*^ is located downstream of *nad5* and *tRNA*^*Glu*^ is located in the non-coding region, separating this region into two parts (Table 2). The same arrangement is seen in other *Paragonimus* species, *Fasciolopsis buski, Fascioloides magna* and *Echinochasmus japonicus*, but these two tRNA genes have exchanged positions in opisthorchiids (*Opisthorchis felineus, O. viverrini* and *Clonorchis sinensis*) and some fasciolids (*Fasciola gigantica, F. hepatica*) (Le et al., 2016; Ma et al., 2015; Ma et al., 2016a; Ma et al., 2017; Shekhovtsov et al., 2010; Cai et al., 2012; Liu et al., 2014b).

### Polymorphism in non-coding regions feature long and short repeats

The major non-coding region (NR) in *P. ohirai* from Kinosaki is 1,465 bp in length and divided into two parts: the short NR (SNR, 328 nucleotides) bounded by *tRNA*^*Gly*^ and *tRNA*^*Glu*^, and the long NR (LNR, 1,137 nucleotides) between *tRNA*^*Glu*^ and *cox3*. There are two long identical repeats (LR1 and LR2, 292 bp each) and six short, identical, tandem repeats (STR1–6) each of 117 bp, in the fully sequenced individual (Pohi-Kinosaki-JP) (Table 2; see GenBank: KX765277). LR1 is located within the SNR while the LR2 overlaps the 3′  end of *tRNA*^*Glu*^by 29 bp at the start of the long non-coding region. STR1–6 immediately follow and a 157-nucleotide unique sequence region occurs between STR6 and *cox3* (Table 2; Fig. S2).

We amplified and sequenced this non-coding region from additional specimens from Kinosaki and Nagoya (Fig. S3) (GenBank: KX765277; MF510407; MG214475 –MG214478). In most cases, their NRs were identical in structure and sequence to that in the fully sequenced specimen from Kinosaki (Pohi-Kinosaki-JP). However, in one individual from Nagoya, this region was 2,602 bp long, 1,404 bp longer than that of the Pohi-Kinosaki-JP sample. This individual from Nagoya (GenBank: MF510407) possessed 18 identical 117-bp STRs, 12 more than in the Pohi-Kinosaki-JP individual (Fig. S3).

Length variability in non-coding regions in most trematodes, including *P. ohirai*, is mainly due to different numbers of repeats, which are common features of this region. This polymorphism, seen also in *P. westermani, Echinochasmus japonicus, Schistosoma* spp. and other trematodes, contributes to a high level of mitochondrial inter-individual and inter-species variation (Blair et al., 2016; Bernt et al., 2013; Le, Blair & McManus, 2001a; Le, Blair & McManus, 2002; Le, Blair & McManus, 2004; Zarowiecki, Huyse & Littlewood, 2007; Le et al., 2016; Oey et al., 2019).

Some caution is required when comparing lengths and structures of non-coding regions from published sources. The long NRs are often not fully represented in mitogenomes in GenBank, despite statements to the contrary. This means that reported lengths of mitogenomes may be very different from actual lengths. When common next-generation sequencing (NGS) methods are used, the presence of multiple and long repeats causes assembly difficulties (Oey et al., 2019). PacBio long-reads were used to establish the structure of the NR in the mitogenome of an Indian *P. westermani* (Oey et al., 2019). This genome was 20.3 kb in length, with 6.3–6.9 kb of repetitive NR region containing three distinct types of repeats of lengths ranging between 229 and 406 bp. Oey et al., 2019 found evidence of length variation, that might occur at the intra-individual level. In BLAST searches, the closest match was with an Indian *P. westermani* Type 1 mitogenome (KM280646: 14,103 bp) that had been assembled from short SOLiD reads. Despite high sequence similarity elsewhere in these two genomes, there was only partial similarity with the repeat motifs.

A further two mitogenomes of *Paragonimus* species have been published recently. These are of *P. kellicotti* (13,927 bp by Wang et al. (2018) and *P. heterotremus* (13,927 bp by Qian et al. (2018). Both were assembled from short-read NGS data: the NR may therefore not be complete. In the NR of *P. kellicotti*, there are three copies (complete or nearly so) of a 111 bp region. In the published genome (MH322000) these are at positions 13,345–13,355, 13,456–13,566 and 13,567–13,362. This repeat does not closely resemble those in other *Paragonimus* species. A putative *tRNA*^*Glu*^, not identified by Wang et al. (2018), starts at 13,289 and ends at 13,348. In the NR of *P. heterotremus* (MH059809) there are three complete or near-complete copies of a 37 bp region (13,503–13,539, 13,578–13,614, 13,686–13,717). Interestingly, this motif is a near-palindrome (with BLAST matches at almost the same locations in the reverse orientation). There are also two copies of a 52 bp region (13,614–13,665 and 13,762–13,813). No similarity was found with other *Paragonimus* species. *tRNA*^*Glu*^ is from 13,343 to 13,408.

**Figure 3:**
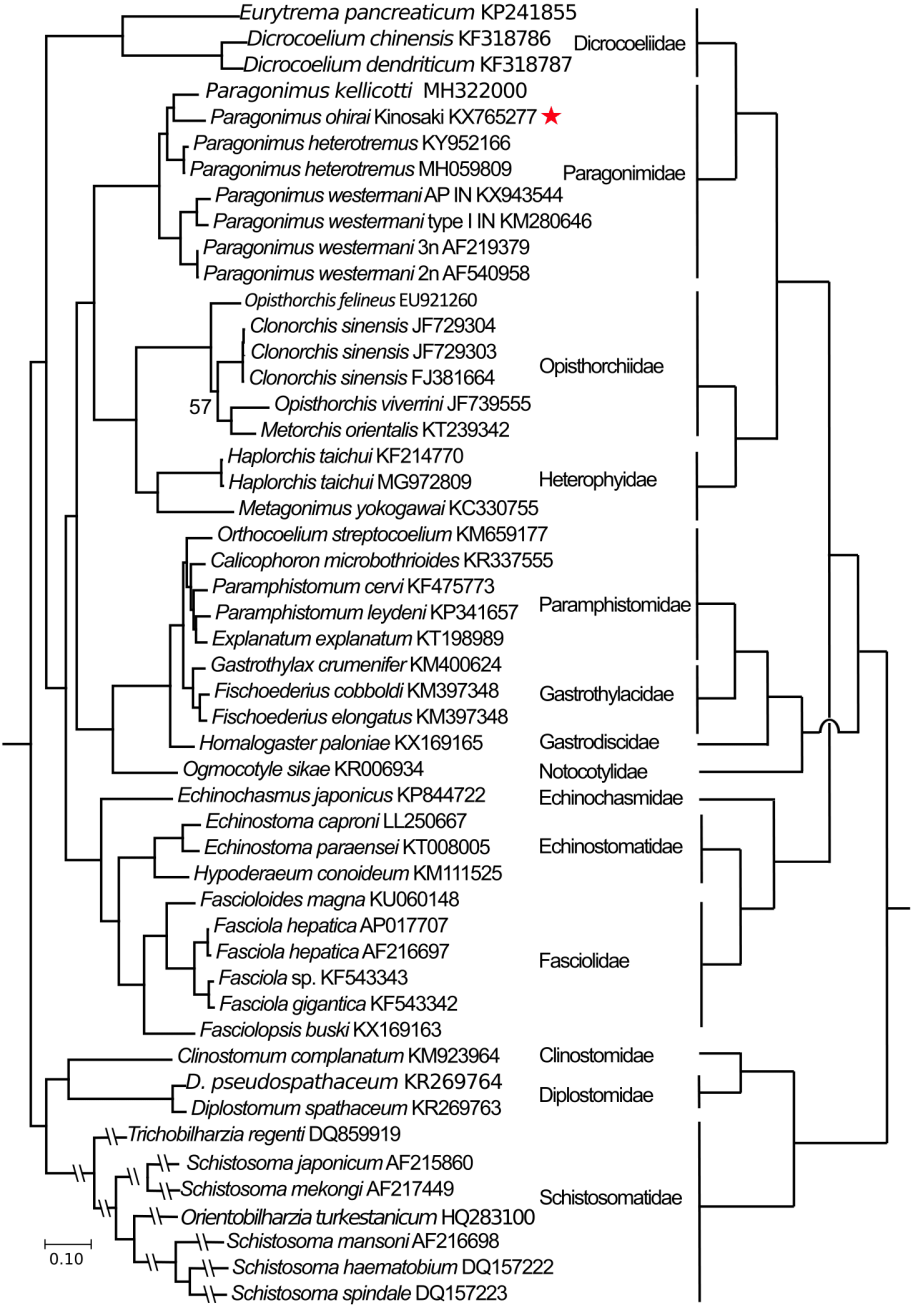
Phylogenetic tree based on concatenated amino-acid sequence data for the 12 mitochondrial proteins from 50 strains of 42 digenean trematode species (Table S1). Phylogenetic reconstruction was performed in MrBayes 3.2 program (Ronquist et al., 2012) with a final alignment of 2,509 amino-acid residues in length after removing poor-quality regions of the alignment. The tree produced (left-hand side of figure) was a 50% majority-rule tree to which all compatible groupings were added and rooted by outgroup (i.e., members of the Diplostomata –schistosomes and related taxa). Bayesian posterior support values for each node were 100% in every case except one (within the Opisthorchiidae, value of 57% indicated). Accession numbers are given at the end of each sequence label. *Paragonimus ohirai* in this study is marked by a star symbol. Full names of each species and of families where they belong are provided. The scale bar represents the number of substitutions per site. The tree on the right-hand side of the figure shows the relationships of the same families according to Olson et al. (2003).

### Phylogenetic analysis

Phylogenetic trees were constructed from concatenated amino-acid sequences from 50 taxa of trematodes (see Table S1). The Bayesian analysis recovered a monophyletic Paragonimidae with the cluster of *P. westermani* sequences appearing as sister to the remaining members of the genus. This matches previous phylogenetic schemes for *Paragonimus* (e.g., Nawa et al., 2014). The two Indian *P. westermani* mitogenomes, originating from the same region of India, despite clustering together, were more distinct from each other than were the two Korean (2n and 3n) mitogenomes (Fig. 3). Three distinct genotypes belonging to the *P. westermani* complex are known from northeastern India (Devi et al., 2010; Devi et al., 2013; Oey et al., 2019). The two genomes of *P. heterotremus* are near-identical. *Paragonimus ohirai* is distinct from the other species, with *P. kellicotti* as a rather distant sister.

The relationships depicted in the Bayesian tree matched reasonably closely those found by Olson et al. (2003) in a study using nuclear ribosomal sequences. Each included family was distinguished and grouped into the suborders proposed in *Olson et al. (2003)* (Fig. 3). For example, fasciolids and echinostomes are sister groups within the Echinostomata, as are paramphistomes and notocotylids (within the Pronocephalata), and also heterophyids and opisthorchiids (within the Opisthorchiata) (Fig. 3). The suborders mostly appear in the tree where expected from *Olson et al. (2003)*, with two striking exceptions. The first is that the dicrocoeliids appear as rather basal in the tree, whereas they should belong close to *Paragonimus* within the derived suborder Xiphidiata (Olson et al., 2003). We can offer no convincing explanation for this discrepancy. The other exception is that, in our tree, the echinostomes and fasciolids (Echinostomata) are basal to the Pronocephalata, a situation reversed in Olson et al. (2003).

## Conclusion

The present study provides the fully annotated mitogenome of *Paragonimus ohirai* (from an individual worm from Kinosaki, Japan) and a description of its genomic features in comparison with those of other paragonimids. There is an emphasis on comparisons with members of the *P. westermani* species complex. Although genomic organization is the same in *P. westermani* and *P. ohirai*, their mitogenomes differ remarkably in base composition, influencing codon usage and skew values. Despite this, phylogenetic analysis of the trematodes recovered a monophyletic family Paragonimidae. Fully characterized mitogenomes of additional paragonimid species will be useful for diagnostic, taxonomic, epidemiological, systematic, phylogenetic and population genetic studies.

##  Supplemental Information

10.7717/peerj.7031/supp-1 Table S1 List of the trematodes for which complete mitochondrial genomes (or at least the coding portion) are available in GenBank Abbr: abbreviation of trematode species name; UNK: unknown; N/A: not available. *Scientific names of hosts, where indicated in original references, are as follows: Cattle/Cow: *Bos taurus*; Human: *Homo sapiens*; Yak: *Bos grunniens*; Goat: *Capra aegagrus hircus*; Cray: Crayfish *Cambarus bouchardi*; Dog: Canis familiaris ; Buffalo: *Bubalus bubalis*; Deer: *Cervus elaphus*; Crabs: *Sesarma dehaani* (Japan), *Barytelphusa lugubris lugubris* (India)*;* Rat: *Rattus norvegicus;* Duck: *Anas platyrhynchos*; Cat: *Felis catus*; Fish: Cyprinoid fish; Black-headed gull: *Larus ridibundus*; Sheep: Ovis aries; culture: a laboratory-maintained strain.

10.7717/peerj.7031/supp-2 Table S2 Codon usage in the mitochondrial genomes of several *Paragonimus* species * Three-letter abbreviations for amino acids according to DDBJ (http://www.ddbj.nig.ac.jp/sub/ref2-e.html). The table cells highlighted indicate examples of bias towards use of A and T (for example, ATT codon for Isoleucine (Ile), TTA for Leucine (Leu), TCT for Serine (Ser), and TAT for Tyrosine (Tyr)).

10.7717/peerj.7031/supp-3 Figure S1 Phylogenetic tree inferred from alignment of partial *cox1* nucleotides The alignment included 224 partial *cox1* nucleotide sequences (309 bp) of 19 *Paragonimus* species available in GenBank and from previous publications, including *Paragonimus ohirai* from three different localities in Japan. The newly sequenced specimen from Kinosaki is indicated by a star symbol. Phylogenetic reconstruction was performed using maximum-likelihood analysis (ML) with the Tamura-Nei model in the MEGA 7 package (*Kumar, Stecher & Tamura, 2016*). Bootstrap support for each node was evaluated using 100 bootstrap resamplings and values reported above the node. Accession numbers and species names are given. The sequences for all species complexes, except the *P. ohirai* complex, have been compressed into triangles. The paraphyletic nature of *P. harinasutai* and *P. bangkokensis* has been noted previously (*(Habe et al., 2013)*). The scale bar indicates the number of substitutions per site.

10.7717/peerj.7031/supp-4 Figure S2 Schematic presentation of the fully annotated sequence (14,818 bp) of the complete mitochondrial genome of *Paragonimus ohirai* (Kinosaki isolate, Japan) The circular molecule contains 12 protein-coding, two ribosomal (large and small subunits), 22 transfer RNA genes and a non-coding region comprising of repeats (Genbank KX765277). For the abbreviations used for individual genes and regions, see Table 3 and Fig. 1. Start and stop codons for each protein-coding gene are highlighted. Repeat sequences are indicated by alternating colour highlights.

10.7717/peerj.7031/supp-5 Figure S3 Sequence alignment of mitochondrial non-coding regions of 6 individuals of *Paragonimus ohirai* The sequences were from 3 Kinosaki (upper lines) and 3 Nagoya specimens (lower lines) obtained by forward primer GLYF (binding in* tRNA*^*Gly*^**) and reverse primer PO17R (located in *cox3*) (Table 1). Location of individual genes and repeats in the aligned region is marked.

## Additional Information and Declarations

### Data Availability

The following information was supplied regarding data availability: Fully annotated mitochondrial genome of *Paragonimus ohirai* (KX765277) and non-coding regions (MF510407; MG214475–MG214478) are available in the GenBank database.
