# Supplementary material for: The complete mitochondrial genome of Paragonimus ohirai (Paragonimidae: Trematoda: Platyhelminthes) and its comparison with P. westermani congeners and other trematodes
Source: PeerJ. 2019 Jun 20;7:e7031. doi: 10.7717/peerj.7031 (PMC6589331; doi:10.7717/peerj.7031)
Supplement: Table S1 — Abbr: abbreviation of trematode species name; UNK: unknown; N/A: not available. *Scientific names of hosts, where indicated in original references, are as follows: Cattle/Cow: Bos taurus; Human: Homo sapiens; Yak: Bos grunniens; Goat: Capra aegagrus hircus; Cray: Crayfish Cambarus bouchardi; Dog: Canis familiaris ; Buffalo: Bubalus bubalis; Deer: Cervus elaphus; Crabs: Sesarma dehaani (Japan), Barytelphusa lugubris lugubris (India); Rat: Rattus norvegicus; Duck: Anas platyrhynchos; Cat: Felis catus; Fish: Cyprinoid fish; Black-headed gull: Larus ridibundus; Sheep: Ovis aries; culture: a laboratory-maintained strain. [file peerj-07-7031-s001.doc]

**Table S1. List of the trematodes for which complete mitochondrial genomes (or at least the coding portion) are available in GenBank**

| **Family** | **Species** | **Abbr** | **Hosts*** | **Geographic origin/**  **Strains** | **Country** | **GenBank**  **Number** | **References** |
| --- | --- | --- | --- | --- | --- | --- | --- |
| **Paragonimidae** | *Paragonimus ohirai* | Pohi | Crab/Rat | Kinosaki | Japan | KX765277 | This study |
|  | *Paragonimus westermani* (2n) | Pwes (2n) | Cray/Dog | Haenam | South Korea | AF540958 | GenBank |
|  | *Paragonimus westermani* (3n) | Pwes (3n) | Cray/Dog | Bogil | South Korea | AF219379 | GenBank |
|  | *Paragonimus westermani* | Pwes | Rat | Type I: in Assam | India | KM280646 | GenBank |
|  | *Paragonimus westermani* | Pwes | Crab | AP: Arunachal Pradesh | India | KX943544 | *Biswal et al., 2014* |
|  | *Paragonimus heterotremus* | Phet_VN | Crab/Dog | Lai Chau | Vietnam | KY952166 | Genbank |
|  | *Paragonimus heterotremus* | Phet_China | Dog | Guanxi | China | MH059809 | *Qian et al., 2018* |
|  | *Paragonimus kellicotti* | Pkell | Cray | Missouri | USA | MH322000 | *Wang et al., 2018* |
| **Clinostomidae** | *Clinostomum complanatum* | Ccom | Fish | Hubei | China | KM923964 | *Chen et al., 2016* |
| **Dicrocoeliidae** | *Dicrocoelium chinensis* | Dchi | Yak | Gansu | China | KF318786 | *Liu et al., 2014a* |
|  | *Dicrocoelium dendriticum* | Dden | Goat | Gansu | China | KF318787 | *Liu et al., 2014a* |
|  | *Eurytrema pancreaticum* | Epan | Cattle | HLJ: Heilongjiang | China | KP241855 | *Chang et al., 2016* |
| **Diplostomidae** | *Diplostomum pseudospathaceum* | Dpse | Black-headed Gull | Tovačov | Czek | KR269764 | *Brabec et al., 2015* |
|  | *Diplostomum spathaceum* | Dspa | Black-headed Gull | Chropyně | Czek | KR269763 | *Brabec et al., 2015* |
| **Echinochasmidae** | *Echinochasmus japonicus* | Ejap | Human | PhuTho | Vietnam | KP844722 | *Le et al., 2016* |
| **Echinostomatidae** | *Echinostoma caproni* | Ecap | UNK | SAMEA: Biosample (SAMEA1034762) | Egypt | AP017706 | GenBank |
|  | *Echinostoma paraensei* | Epar | UNK | UNM | Mexico | KT008005 | GenBank |
|  | *Hypoderaeum conoideum* | Hcon | Duck | Hubei | China | KM111525 | *Yang et al., 2015a* |
| **Fasciolidae** | *Fasciola gigantica* | Fgig | Buffalo | Guangxi | China | KF543342 | *Liu et al., 2014b* |
|  | *Fasciola* sp, intermediate form | Fsp | Cow | GHL2014: Heilongjiang | China | KF543343 | *Liu et al., 2014b* |
|  | *Fasciola hepatica* | Fhep | Cattle | Geelong | Australia | AF216697 | *Le, Blair & McManus, 2001a* |
|  | *Fasciola hepatica* | Fhep | N/A | Submitted by Japan (byJP) | UNK | AP017707 | GenBank |
|  | *Fascioloides magna* | Fmag | Deer | Kokořínsko | Czech | KU060148 | *Ma et al., 2016a* |
|  | *Fasciolopsis buski* | Fbus | Pig | Jiangxi | China | KX169163 | *Ma et al., 2017* |
| **Gastrodiscidae** | *Homalogaster paloniae* | Hpal | Goat | Hubei | China | KX169165 | *Yang et al., 2016a* |
| **Gastrothylacidae** | *Fischoederius cobboldi* | Fcob | UNK | N/A | China | KX169164 | GenBank |
|  | *Fischoederius elongatus* | Felo | Cattle | Hubei | China | KM397348 | *Yang et al., 2015b* |
|  | *Gastrothylax crumenifer* | Gcru | Cattle | Hubei | China | KM400624 | *Yang et al., 2016b* |
| **Heterophyidae** | *Haplorchis taichui* | Htai | Human | N/A | Laos | KF214770 | *Lee et al., 2013* |
|  |  | Htai | Human | QuangTri | Vietnam | MG972809 | GenBank |
|  | *Metagonimus yokogawai* | Myok | UNK | N/A | South Korea | KC330755 | GenBank |
| **Notocotylidae** | *Ogmocotyle sikae* | Osik | Goat | OHX-Hunan | China | KR006934 | Ma et al., 2016b |
| **Opisthorchiidae** | *Clonorchis sinensis* | Csin | Fish | Amur: river in Novosibirsk | Russia | FJ381664 | *Shekhovtsov et al., 2010* |
|  |  | Csin | Cat | Gdong: Guangdong | China | JF729303 | *Cai et al., 2012* |
|  |  | Csin | Cat | N/A | South Korea | JF729304 | *Cai et al., 2012* |
|  | *Metorchis orientalis* | Mori | Duck | HLJ: Heilongjiang | China | KT239342 | *Na et al., 2019* |
|  | *Opisthorchis felineus* | Ofel | Cat | UstTula: in Novosibirsk | Russia | EU921260 | *Shekhovtsov et al., 2010* |
|  | *Opisthorchis viverrini* | Oviv | Cat | N/A | Laos | JF739555 | *Cai et al., 2012* |
| **Paramphistomidae** | *Calicophoron microbothrioides* | Cmic | Human | Hunan | China | KR337555 | GenBank |
|  | *Explanatum explanatum* | Eexp | UNK | Hunan | China | KT198989 | GenBank |
|  | *Paramphistomum cervi* | Pcer | Yak | Qinghai | China | KF475773 | *Yan et al., 2013* |
|  | *Orthocoelium streptocoelium* | Ostr | Cattle | Tianmen | China | KM659177 | *Zhao et al., 2016* |
|  | *Paramphistomum leydeni* | Pley | Goat | Nimu: in Tibet Autonomous Region | China | KP341657 | *Ma et al., 2015* |
| **Schistosomatidae** | *Orientobilharzia turkestanicum* | Otur | Sheep | HLJ: Heilongjiang | China | HQ283100 | *Wang et al., 2011* |
|  | *Schistosoma haematobium* | Shae | culture | N10: a village in Mali | Mali | DQ157222 | *Littlewood et al., 2006* |
|  | *Schistosoma japonicum* | Sjap | culture | Anhui | China | AF215860 | *Le et al., 2001b* |
|  | *Schistosoma mansoni* | Sman | culture | NMRI | Puerto Rico | AF216698 | *Le et al., 2001b* |
|  | *Schistosoma mekongi* | Smek | culture | Khong | Laos | AF217449 | *Le et al., 2001b* |
|  | *Schistosoma spindale* | Sspi | culture | WWBL | Sri Lanka | DQ157223 | *Littlewood et al., 2006* |
|  | *Trichobilharzia regenti* | Treg | UNK | N/A | N/A | DQ859919 | *Webster et al., 2007* |

Abbr: abbreviation of trematode species name; UNK: unknown; N/A: not available.

*Scientific names of hosts, where indicated in original references, are as follows: Cattle/Cow: *Bos taurus*; Human: *Homo sapiens*; Yak: *Bos grunniens*; Goat: *Capra aegagrus hircus*; Cray: Crayfish *Cambarus bouchardi*; Dog: Canis familiaris; Buffalo: *Bubalus bubalis*; Deer: *Cervus elaphus*; Crabs: *Sesarma dehaani* (Japan), *Barytelphusa lugubris lugubris* (India)*;* Rat: *Rattus norvegicus;* Duck: *Anas platyrhynchos*; Cat: *Felis catus*; Fish: Cyprinoid fish; Black-headed gull: *Larus ridibundus*; Sheep: Ovis aries; culture: a laboratory-maintained strain.
