# Supplementary material for: The complete mitochondrial genome of Paragonimus ohirai (Paragonimidae: Trematoda: Platyhelminthes) and its comparison with P. westermani congeners and other trematodes
Source: PeerJ. 2019 Jun 20;7:e7031. doi: 10.7717/peerj.7031 (PMC6589331; doi:10.7717/peerj.7031)
Supplement: Table S2 — * Three-letter abbreviations for amino acids according to DDBJ (http://www.ddbj.nig.ac.jp/sub/ref2-e.html). The table cells highlighted indicate examples of bias towards use of A and T (for example, ATT codon for Isoleucine (Ile), TTA for Leucine (Leu), TCT for Serine (Ser), and TAT for Tyrosine (Tyr)). [file peerj-07-7031-s002.docx]

**Table S2.** **Codon usage in the mitochondrial genomes of several *Paragonimus* species**

| **Amino acid*** | **Codon** | ***P*. *ohirai* (Japan) KX765277** | | ***P. westermani* (2n) (S. Korea) AF540958** | | ***P. westermani* (3n) (S. Korea) AF219379** | | ***P. westermani* (Type 1) (India) KM280646** | | ***P. westermani* (AP) (India) KX943544** | | ***P. kellicotti* (USA) MH322000** | | ***P. heterotremus* (China) MH059809** | |
| --- | --- | --- | --- | --- | --- | --- | --- | --- | --- | --- | --- | --- | --- | --- | --- |
|  |  | **No** | **%** | **No** | **%** | **No** | **%** | **No** | **%** | **No** | **%** | **No** | **%** | **No** | **%** |
| **Ala** | GCG | 28 | 0.83 | 51 | 1.51 | 50 | 1.49 | 48 | 1.43 | 49 | 1.45 | 44 | 1.31 | 38 | 1.13 |
|  | GCA | 23 | 0.68 | 13 | 0.39 | 12 | 0.36 | 15 | 0.45 | 12 | 0.36 | 13 | 0.39 | 18 | 0.53 |
|  | GCT | 66 | 1.96 | 59 | 1.75 | 61 | 1.81 | 61 | 1.81 | 60 | 1.78 | 67 | 1.99 | 64 | 1.90 |
|  | GCC | 18 | 0.53 | 29 | 0.86 | 29 | 0.86 | 26 | 0.77 | 27 | 0.8 | 18 | 0.53 | 22 | 0.65 |
| **Cys** | TGT | 94 | 2.79 | 70 | 2.08 | 72 | 2.14 | 79 | 2.35 | 84 | 2.49 | 80 | 2.38 | 87 | 2.58 |
|  | TGC | 12 | 0.36 | 39 | 1.16 | 38 | 1.13 | 29 | 0.86 | 24 | 0.71 | 22 | 0.65 | 18 | 0.53 |
| **Asp** | GAT | 56 | 1.66 | 60 | 1.78 | 60 | 1.78 | 55 | 1.63 | 54 | 1.6 | 56 | 1.66 | 59 | 1.75 |
|  | GAC | 10 | 0.3 | 6 | 0.18 | 6 | 0.18 | 12 | 0.36 | 14 | 0.41 | 9 | 0.27 | 9 | 0.27 |
| **Glu** | GAG | 65 | 1.93 | 68 | 2.02 | 68 | 2.02 | 61 | 1.81 | 65 | 1.92 | 68 | 2.02 | 64 | 1.90 |
|  | GAA | 12 | 0.36 | 13 | 0.39 | 13 | 0.39 | 18 | 0.53 | 18 | 0.53 | 10 | 0.30 | 15 | 0.45 |
| **Phe** | TTT | 325 | 9.65 | 208 | 6.19 | 208 | 6.18 | 255 | 7.57 | 253 | 7.49 | 325 | 9.66 | 301 | 8.94 |
|  | TTC | 36 | 1.07 | 130 | 3.9 | 131 | 3.9 | 93 | 2.76 | 82 | 2.43 | 30 | 0.89 | 45 | 1.34 |
| **Gly** | GGG | 88 | 2.61 | 107 | 3.18 | 105 | 3.12 | 103 | 3.06 | 110 | 3.25 | 111 | 3.30 | 99 | 2.94 |
|  | GGA | 68 | 2.02 | 43 | 1.28 | 43 | 1.28 | 58 | 1.72 | 48 | 1.42 | 38 | 1.13 | 37 | 1.10 |
|  | GGT | 132 | 3.92 | 128 | 3.8 | 130 | 3.86 | 109 | 3.24 | 116 | 3.42 | 137 | 4.07 | 146 | 4.34 |
|  | GGC | 19 | 0.56 | 43 | 1.28 | 42 | 1.25 | 39 | 1.16 | 36 | 1.07 | 23 | 0.68 | 36 | 1.07 |
| **His** | CAT | 55 | 1.63 | 32 | 0.95 | 33 | 0.98 | 36 | 1.07 | 37 | 1.1 | 51 | 1.52 | 49 | 1.46 |
|  | CAC | 3 | 0.09 | 28 | 0.83 | 27 | 0.8 | 28 | 0.83 | 26 | 0.77 | 10 | 0.30 | 10 | 0.30 |
| **Ile** | ATA | 37 | 1.1 | 22 | 0.65 | 21 | 0.63 | 22 | 0.65 | 19 | 0.56 | 36 | 1.07 | 26 | 0.77 |
|  | ATT | 137 | 4.07 | 57 | 1.69 | 55 | 1.63 | 73 | 2.17 | 71 | 2.1 | 108 | 3.21 | 101 | 3.00 |
|  | ATC | 11 | 0.33 | 47 | 1.4 | 48 | 1.43 | 34 | 1.01 | 38 | 1.12 | 17 | 0.51 | 25 | 0.74 |
| **Lys** | AAG | 53 | 1.57 | 51 | 1.52 | 50 | 1.49 | 47 | 1.4 | 49 | 1.45 | 47 | 1.40 | 51 | 1.51 |
| **Leu** | TTG | 263 | 7.81 | 232 | 6.89 | 233 | 6.92 | 245 | 7.27 | 252 | 7.46 | 273 | 8.11 | 264 | 7.84 |
|  | TTA | 162 | 4.81 | 35 | 1.04 | 34 | 1.01 | 65 | 1.93 | 60 | 1.78 | 111 | 3.30 | 99 | 2.94 |
|  | CTG | 14 | 0.42 | 148 | 4.4 | 148 | 4.4 | 95 | 2.82 | 92 | 2.72 | 42 | 1.25 | 59 | 1.75 |
|  | CTA | 18 | 0.53 | 13 | 0.39 | 12 | 0.36 | 17 | 0.51 | 18 | 0.53 | 23 | 0.68 | 18 | 0.53 |
|  | CTT | 118 | 3.5 | 80 | 2.38 | 80 | 2.38 | 106 | 3.15 | 105 | 3.11 | 111 | 3.30 | 122 | 3.62 |
|  | CTC | 7 | 0.21 | 71 | 2.11 | 72 | 2.14 | 42 | 1.25 | 45 | 1.33 | 21 | 0.62 | 26 | 0.77 |
| **Met** | ATG | 92 | 2.73 | 85 | 2.53 | 87 | 2.59 | 87 | 2.58 | 86 | 2.54 | 91 | 2.70 | 93 | 2.76 |
| **Asn** | AAA | 18 | 0.53 | 26 | 0.77 | 26 | 0.77 | 27 | 0.8 | 27 | 0.8 | 24 | 0.71 | 22 | 0.65 |
|  | AAT | 51 | 1.51 | 26 | 0.77 | 24 | 0.71 | 32 | 0.95 | 35 | 1.04 | 38 | 1.13 | 41 | 1.22 |
|  | AAC | 6 | 0.18 | 20 | 0.59 | 20 | 0.6 | 19 | 0.56 | 20 | 0.59 | 9 | 0.27 | 11 | 0.33 |
| **Pro** | CCG | 20 | 0.59 | 26 | 0.77 | 27 | 0.8 | 27 | 0.8 | 26 | 0.77 | 23 | 0.68 | 23 | 0.68 |
|  | CCA | 11 | 0.33 | 6 | 0.18 | 5 | 0.15 | 5 | 0.15 | 5 | 0.15 | 6 | 0.18 | 13 | 0.39 |
|  | CCT | 51 | 0.51 | 44 | 1.31 | 41 | 1.22 | 39 | 1.16 | 40 | 1.18 | 46 | 1.37 | 48 | 1.43 |
|  | CCC | 10 | 0.3 | 14 | 0.42 | 18 | 0.54 | 18 | 0.53 | 19 | 0.56 | 18 | 0.53 | 8 | 0.24 |
| **Gln** | CAG | 25 | 0.74 | 25 | 0.74 | 25 | 0.74 | 24 | 0.71 | 24 | 0.71 | 24 | 0.71 | 25 | 0.74 |
|  | CAA | 6 | 0.18 | 3 | 0.09 | 3 | 0.09 | 6 | 0.18 | 4 | 0.12 | 5 | 0.15 | 5 | 0.15 |
| **Arg** | CGG | 13 | 0.39 | 28 | 0.83 | 28 | 0.83 | 23 | 0.68 | 23 | 0.68 | 11 | 0.33 | 16 | 0.48 |
|  | CGA | 7 | 0.21 | 4 | 0.12 | 5 | 0.15 | 9 | 0.27 | 10 | 0.3 | 9 | 0.27 | 3 | 0.09 |
|  | CGT | 42 | 1.25 | 27 | 0.8 | 27 | 0.8 | 29 | 0.86 | 30 | 0.89 | 39 | 1.16 | 38 | 1.13 |
|  | CGC | 1 | 0.03 | 11 | 0.33 | 11 | 0.33 | 6 | 0.18 | 7 | 0.21 | 5 | 0.15 | 7 | 0.21 |
| **Ser** | AGG | 70 | 2.08 | 91 | 2.7 | 88 | 2.61 | 78 | 2.32 | 82 | 2.43 | 70 | 2.08 | 76 | 2.26 |
|  | AGA | 35 | 1.04 | 16 | 0.48 | 18 | 0.54 | 39 | 1.16 | 37 | 1.1 | 31 | 0.92 | 33 | 0.98 |
|  | AGT | 64 | 1.9 | 42 | 1.25 | 44 | 1.31 | 55 | 1.63 | 55 | 1.63 | 63 | 1.87 | 50 | 1.49 |
|  | AGC | 10 | 0.33 | 26 | 0.77 | 25 | 0.74 | 17 | 0.51 | 19 | 0.56 | 20 | 0.59 | 19 | 0.56 |
|  | TCG | 17 | 0.51 | 50 | 1.49 | 49 | 1.46 | 34 | 1.01 | 37 | 1.1 | 26 | 0.77 | 35 | 1.04 |
|  | TCA | 19 | 0.56 | 7 | 0.21 | 6 | 0.18 | 16 | 0.48 | 14 | 0.41 | 11 | 0.33 | 3 | 0.09 |
|  | TCT | 137 | 4.07 | 79 | 2.35 | 80 | 2.38 | 99 | 2.94 | 105 | 3.11 | 128 | 3.80 | 121 | 3.59 |
|  | TCC | 14 | 0.42 | 52 | 1.54 | 51 | 1.52 | 34 | 1.01 | 41 | 1.21 | 24 | 0.71 | 22 | 0.65 |
| **Thr** | ACG | 22 | 0.65 | 37 | 1.1 | 36 | 1.07 | 36 | 1.07 | 37 | 1.1 | 22 | 0.65 | 26 | 0.77 |
|  | ACA | 14 | 0.42 | 4 | 0.12 | 3 | 0.09 | 6 | 0.18 | 4 | 0.12 | 6 | 0.18 | 5 | 0.15 |
|  | ACT | 42 | 1.25 | 25 | 0.74 | 26 | 0.77 | 37 | 1.1 | 33 | 0.98 | 47 | 1.40 | 42 | 1.25 |
|  | ACC | 4 | 0.12 | 18 | 0.54 | 20 | 0.59 | 11 | 0.33 | 9 | 0.27 | 14 | 0.42 | 10 | 0.30 |
| **Val** | GTG | 92 | 2.73 | 166 | 4.93 | 169 | 5.2 | 158 | 4.69 | 159 | 4.7 | 110 | 3.27 | 121 | 3.59 |
|  | GTA | 44 | 1.31 | 15 | 0.45 | 17 | 0.51 | 26 | 0.77 | 25 | 0.74 | 32 | 0.95 | 20 | 0.59 |
|  | GTT | 205 | 6.09 | 153 | 4.54 | 152 | 4.51 | 183 | 5.43 | 180 | 5.33 | 219 | 6.51 | 211 | 6.27 |
|  | GTC | 23 | 0.68 | 85 | 2.53 | 83 | 2.47 | 43 | 1.28 | 45 | 1.33 | 18 | 0.53 | 37 | 1.10 |
| **Trp** | TGG | 56 | 1.66 | 91 | 2.7 | 91 | 2.7 | 86 | 2.55 | 83 | 2.46 | 89 | 2.64 | 75 | 2.23 |
|  | TGA | 55 | 1.63 | 20 | 0.59 | 20 | 0.59 | 31 | 0.92 | 34 | 1.01 | 22 | 0.65 | 39 | 1.16 |
| Tyr | TAT | 137 | 4.07 | 48 | 1.43 | 47 | 1.4 | 77 | 2.29 | 79 | 2.34 | 130 | 3.86 | 116 | 3.45 |
|  | TAC | 13 | 0.39 | 102 | 3.03 | 102 | 3.03 | 68 | 2.02 | 70 | 2.07 | 23 | 0.68 | 33 | 0.98 |
| stop | TAG | 11 | 0.33 | 11 | 0.33 | 11 | 0.33 | 10 | 0.3 | 9 | 0.27 | 11 | 0.33 | 11 | 0.33 |
|  | TAA | 1 | 0.03 | 1 | 0.03 | 1 | 0.03 | 2 | 0.06 | 3 | 0.09 | 1 | 0.03 | 1 | 0.03 |
| * Three-letter abbreviations for amino acids according to DDBJ (http://www.ddbj.nig.ac.jp/sub/ref2-e.html). The table cells highlighted indicate examples of bias towards use of A and T (for example, ATT codon for Isoleucine (Ile), TTA for Leucine (Leu), TCT for Serine (Ser), and TAT for Tyrosine (Tyr)) . | | | | | | | | | | | | | | | |
