# Supplementary figures and images for: The complete mitochondrial genome of Paragonimus ohirai (Paragonimidae: Trematoda: Platyhelminthes) and its comparison with P. westermani congeners and other trematodes
Source: PeerJ. 2019 Jun 20;7:e7031. doi: 10.7717/peerj.7031 (PMC6589331; doi:10.7717/peerj.7031)

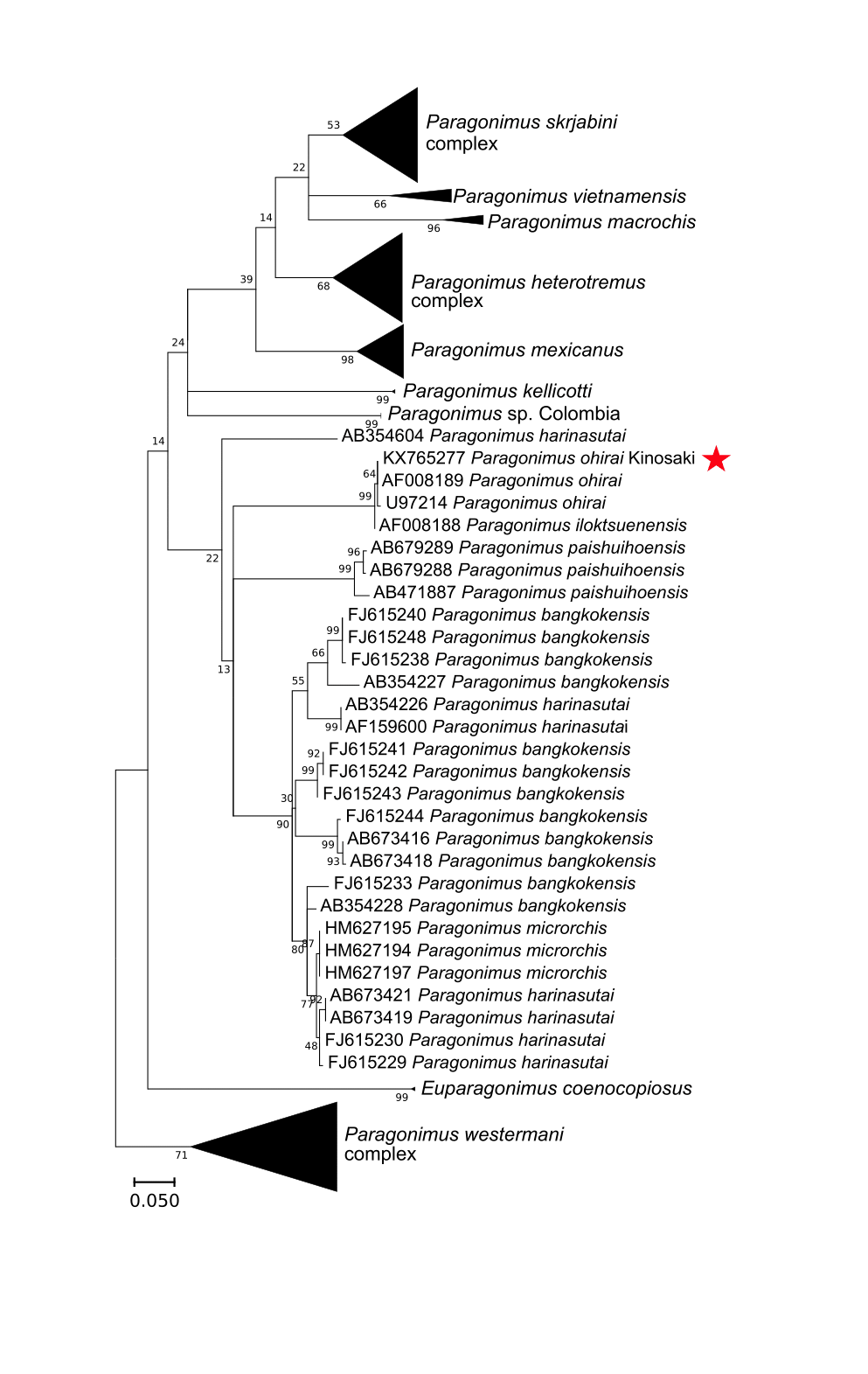

Supplement: Figure S1 — The alignment included 224 partial cox1 nucleotide sequences (309 bp) of 19 Paragonimus species available in GenBank and from previous publications, including Paragonimus ohirai from three different localities in Japan. The newly sequenced specimen from Kinosaki is indicated by a star symbol. Phylogenetic reconstruction was performed using maximum-likelihood analysis (ML) with the Tamura-Nei model in the MEGA 7 package (Kumar, Stecher & Tamura, 2016). Bootstrap support for each node was evaluated using 100 bootstrap resamplings and values reported above the node. Accession numbers and species names are given. The sequences for all species complexes, except the P. ohirai complex, have been compressed into triangles. The paraphyletic nature of P. harinasutai and P. bangkokensis has been noted previously ((Habe et al., 2013)). The scale bar indicates the number of substitutions per site. [file peerj-07-7031-s003.png]
