# Supplementary material for: The complete mitochondrial genome of Paragonimus ohirai (Paragonimidae: Trematoda: Platyhelminthes) and its comparison with P. westermani congeners and other trematodes
Source: PeerJ. 2019 Jun 20;7:e7031. doi: 10.7717/peerj.7031 (PMC6589331; doi:10.7717/peerj.7031)
Supplement: Figure S2 — The circular molecule contains 12 protein-coding, two ribosomal (large and small subunits), 22 transfer RNA genes and a non-coding region comprising of repeats (Genbank KX765277). For the abbreviations used for individual genes and regions, see Table 3 and Fig. 1. Start and stop codons for each protein-coding gene are highlighted. Repeat sequences are indicated by alternating colour highlights. [file peerj-07-7031-s004.pdf]

```
cox3        *        20         *        40         *        60         *        80         *       100         *       120         *       140         *       160         *
Pohi(14818 : ATGAGATGATTACCTTTGTATAATTCGTGGTTGTTTTTTATGGCTCTTTTAAGGTTTTTTCTTTGAAAGATTGTAGGTGTTTTCTCTCTTTTTTGTTTAGTGGGACTTTCTGTTTTTATGTTGATTAGTGAGGTAATGCCTTATAAGGTTCATTATGAGCTTGGATTTTG :   170
        180         *       200         *       220         *       240         *       260         *       280         *       300         *       320         *       340
Pohi(14818 : GCTTTTTATTCTTAGGGAGGTTGCCATTTTTGGTACTTTGTTTGTAATGTGTCTTTGGAGGATGGATGGAGCGACGGAGTCGATTTCTAGGGCAACGGAGCTTCCACTTTTAGGATCATTTTTATTGATTGGTTCTTCAATTACGGCGACTACATATCATCATTGTCGGG :   340
          *       360         *       380         *       400         *       420         *       440         *       460         *       480         *       500         *
Pohi(14818 : GTTTGAATATTAGTTGGGTGTTTTTGGCGGTTACTATTGTTCTTGGGTTGAGTTTTATCCTATTGCAGGCTGTTGAGTTTTATGAATGTGAGTGTGATGTTTTAAAAGATGTGTATTATTCTAGTGTTTTTTCTACGGTGGGCTTGCATTTTAGTCATGTTTTTGTAGGG :   510
        520         *       540         *       560         *       580         *       600         *       620         *       640         * trnH   660         *       680
Pohi(14818 : GTGTTGGGTTTATCTGTTTTAATGTTGTTGGGGGCTAATACGGTTAAGCAGTGTTATGCTAAAGTTGCTGTGTGGTATTGGCATTTTGTTGATTATGTGTGGTTGTTTGTTTTTTTCTTGTTATATTTTTGTAGATTTCCTCCCTAGGTTAATTTTTTTAAACCATTGG :   680
          *       700         *       720 cob         *       740         *       760         *       780         *       800         *       820         *       840         *
Pohi(14818 : TTTGTGGGGCCGGGGATCTAATATTTTAGGGGCGGAAATGTTATCTGTGTTGCGTTCTAAAGTTGTGGATTTACCTACAAATTTGTCTTTGAGATATCTTTGATGTGGAGGGTTTATGATCAGAAGGTTTTTAGTTCTTCAGTTAGGTTCTGGTGTGGTGTTGTCGTTGC :   850
        860         *       880         *       900         *       920         *       940         *       960         *       980         *      1000         *      1020
Pohi(14818 : TTTATGTGGCTGATGCTGGGTTGAGGTTTGGTTGTGTTTTAGATTTTACTGGGGAGAGTGTTTTTGTTTGGCTAATTCGTTATCTTCATATTTGAGGGGTTACTTTTATTTTATCTTGTTTTTTATGCATATGGGTCGGGCCTTGTATTATTCTAGTTATAGGAAGGTG :  1020
          *      1040         *      1060         *      1080         *      1100         *      1120         *      1140         *      1160         *      1180         *
Pohi(14818 : GCGGTTTGAAATGTTGGGTTCATTTTATATCTTTTGATGATGATTGAGGCATTTTTAGGGTATATATTGCCTTGACATCAGATGTCTTACTGGGCTGCGACGGTTTTAACGTCAATTCTTATAGGGTTCCTTTAATTGGGCCCAGCTTGTACACTTTATAGTTGGTGG :  1190
       1200         *      1220         *      1240         *      1260         *      1280         *      1300         *      1320         *      1340         *      1360
Pohi(14818 : ATTTGGTGTAACAAATGCTACGTTGGTTCGGGTGTTTTCTGCGCATGTTTGTTTGGCTTTTATCATTGTGGGTTTTAGCATTATACATCTTTTTACCTGCATAAGGGTGGTTCTAATAATCCATTGTTTGTGAGAGGGGGGTATAGTGATGTGGTTTTATTTCATAGAT :  1360
          *      1380         *      1400         *      1420         *      1440         *      1460         *      1480         *      1500         *      1520         *
Pohi(14818 : TTTTTACTAATAAGGACGGTTAATGTTAATGATTCTTTTGAGATTACTTGGGGGATTTATGTTAATTTGTCCGGATTTGGTATTAGATGTGAGAGATATTTGCAGGCGGATCCTTTGGTAACGCCCGTTTCAATAAAGCCGGAGTGATATTTTTGGCTTTTTATGCT :  1530
       1540         *      1560         *      1580         *      1600         *      1620         *      1640         *      1660         *      1680         *      1700
Pohi(14818 : ATGCTTCGTTCTATTGAGTCTAAGGTTGGGGGTTTAATTTTGGTGTTGGTTTTTTTATTTATTCTTGGTTGCCGAGGTTTAATAATTCTTGTTTATTTTTGGGTCGTCAGTACATTTTTTAGGTGCTTCGTCGATGTTTTTCTTCTTAGGTATTTAGGAGCTTG :  1700
          *      1720         *      1740         *      1760         *      1780         *      1800         *      1820         *      1840 nad4L      *      1860         *
Pohi(14818 : TCATCCTGAGGATCCATATATTTGATTAGTAAGCTTTGTAGGTTGTTTATTGTTCTTTTTCTGTTTTTGTTTAAGGGATTGTGAACAGTGCCTTATTCTTTTGGTGTTCCTTTTAGGGGAGTAAAGGCCTTTTAGTTAGTATGGGGGTGAGTGTTTTGTTTTTAGGTGG :  1870
       1880         *      1900         *      1920         *      1940         *      1960         *      1980         *      2000         *      2020         *      2040
Pohi(14818 : TGTAGTTGTGTTGTGTGGCCTTGTTTtATCATTGTCTCGTTTTTAAATTGTTTAATTATCGTTGAAAAATTTAACGTGTTGTTGCTTTTTCTTCGATATTGCAGCAATGACAGGAGAGACATATTTGTTTATTGCCTTAATGGTAATTTTACTGTTGAGGTTGTTT :  2040
          *      2060 nad4 *      2080         *      2100         *      2120         *      2140         *      2160         *      2180         *      2200         *
Pohi(14818 : TGGGCTTAGTGGTGCTTACTCGATTATGAGACTTGGAAGTTTGATTGGATTAGTTGGTTGATAGGTATTGGGATTGTTTTTTTTTTACATGTTGTTGTGGGTGGTGTTTGTCTTCTCCTCTTTTAGGGGGGTTTGAGTGGACAGGTTTTTTGTTTTGATATCTTGAGCTTTT :  2210
       2220         *      2240         *      2260         *      2280         *      2300         *      2320         *      2340         *      2360         *      2380
Pohi(14818 : ATCTGGAGGCCTGTCCTTAATATTAGCGGTGTCTTTGGTTTTCTGACAGAGGTATTTTAGGGGGGTTTCTCGTTTTATGATTTTTTTGAGGTTAGTCTCTCTTTGTTTAGGTATTGTTGTGTCCATTCTTTAGGATTTTGGGTTTTTTACGAGGGTGCAATATTGCCT :  2380
          *      2400         *      2420         *      2440         *      2460         *      2480         *      2500         *      2520         *      2540         *
Pohi(14818 : CTTTTGTTTTTTATTAATTCGGGAGTCTCCTTATTCAGAGCGTTATGTGGCGGCGTGATATTTGCTGGGGTATGTAATTTTGACAAGGCTTCCAATGTTGTTGTGTTTTATCTATTTTTCTTGTGAGGCAGGTAGATTTAATGTACGTTATTGGGAGGAGGCTTCCGTAGG :  2550
       2560         *      2580         *      2600         *      2620         *      2640         *      2660         *      2680         *      2700         *      2720
Pohi(14818 : GGGCTTTAGGGTTATTTTATTGCTATCTGTGTTGTTTATAACTAAGATTCCTTTGCCTCCATTTCATGTTTGGTTGCCGATTGTTCATGCTGAGGCTAGAAGGCCGGTTTCTGTTTGTTTGAGAGGTTAGCCATATAATGAAGTTGGGGTTACTAGGTGTTTATCGTTTCTGTT :  2720
          *      2740         *      2760         *      2780         *      2800         *      2820         *      2840         *      2860         *      2880         *
Pohi(14818 : TTTGATTACTTCCGAGGTATATCTTTTCTGTGCCTTATGTAGGTATTGGGGTTTTGATGACGTTGCTATTTTTCTTTAGTGCAGCTCGAGAATTGGATGGGAAGCGTTGATTGGCGTTTATGAGATTAGCCCATATTTATTGATTCCTGCAGTGTGTCTTTGTGTTAGTGGC :  2890
       2900         *      2920         *      2940         *      2960         *      2980         *      3000         *      3020         *      3040         *      3060
Pohi(14818 : TTTGATAATTTAGGTATTTCCTTTTTGTATTGTTTAGGGCATGGTGCTTCAGCTGGTGTTATGTTTCTTTTTTTGTGGCTTGCCTACGAGGTATCTGGTAGTCGTAATTGAGCTGTTTTGAAGTTTGGTCTTTCTAATAGTTTGTTGATGCGCTTCTTAGTTGGAGCATG :  3060
          *      3080         *      3100         *      3120         *      3140         *      3160         *      3180         *      3200         *      3220         *
Pohi(14818 : TCTTTGCACTGTCGCTTCTTTACCTCCGACGGTACAGTTTTTTTGTGAGGTTCATACTTTGTGGGAGGCTGGGCCACTGCTTTTAAATTTGGTTTTTTGGTCTTTTTGCTATCTCTTTTGTGGTGGTCTGGTACCGGTTTTTACGTTGGGTGGGTTGTTTTCTGTCATT :  3230
       3240         *      3260         *      3280         *      3300         *      3320         *      3340 trnQ    *      3360         *      3380         *      3400
Pohi(14818 : ATAGTATTGGGTTTAGTGTAAGGCCTATTTTTATGGGATTGTTTTCTGTGTTGTCTTTATTATTTTGAAGGTTTACTTTATTTTTAGTTGGGTAGCTTTATTTATGTGCAGCTCAGTGTTTGTTGTGCATTTTATGTTTTGGTCATAGAGGGGTTTGTTACTAGCTGCAGTT :  3400
          * trnF 3420         *      3440         *      3460         * trnM 3480         *      3500         *      3520         *      3540 atp6      *      3560         *
Pohi(14818 : TAGACTCTCTTCTAGCTTATTTGAAAGCATTAGTTTGAAGAGCTGGGGAAATTAATTATTTATGGGAGAGGGGGGAGGTAAGTTAAGTTAATAAACTGTATGGCTCATGCCTATGTAATACATTTTGTCCTCCCCTATGTATGTGACTCGTTTAGGAAGCATTGGAAGTT :  3570
       3580         *      3600         *      3620         *      3640         *      3660         *      3680         *      3700         *      3720         *      3740
Pohi(14818 : TTTAGTGGGCTTAATATCTGGTGGACAAGGCGATTTTATTTATCGTGTTGTGTTGTGTGGTTTGCTTTTTTGTTTTTTAAGTCTTCGGTTTCTTATCTCTTTGGTTTAAGAGGATTTGGCTTGTTTTTGTTTTTTGTGATTTCTCCCTTATTCTTATCTTTGTTCTTCT :  3740
          *      3760         *      3780         *      3800         *      3820         *      3840         *      3860         *      3880         *      3900         *
Pohi(14818 : CTCGATTTAGAAATGGGGGAGGTCTCCTTTTTTTTTCTTGTTTCGTACCTGAGGGGACTCCTCTTTGAATAGCCCCCTTCGTTTGTATGGCGGAGACTTTAAGTTATTTGGTTCGTCCTGTAGTTTTAATGATTCGTCCCTTTGTTAATTTGTCAATGGGCGCCATGGGA :  3910
       3920         *      3940         *      3960         *      3980         *      4000         *      4020         *      4040         * nad2 4060         *      4080
Pohi(14818 : GGATTTGTTTTGGGTTTGCTTAGGTTTCGTTTTGAGTGGGTGGTTTTTTTTTTCGTTTTTCCTCTTTTTTTATGAGGTCTTTGTTGCGGTTGTGCATTGATTTATAGTTTGTAACATTTTGTCTTTTTCTGAGAGTCATTAGTTATGTGGGGATATTTGTTAGGAATTGTT :  4080
          *      4100         *      4120         *      4140         *      4160         *      4180         *      4200         *      4220         *      4240         *
Pohi(14818 : AGTTTGTTAGGGGTTTTTATTTTTTTGTTTTATTTTTAAGGGATGGTTTGGCAATGTTTTGATTGTTTTTAGAGCTAGGTGCTTTGTCTTTAATTCCTTCATTTTTTTGCGTTTAGGGGATGGTGTTTTGGAAGCTTGTTTAATTATTTAATCGTTCTAGGGT :  4250
       4260         *      4280         *      4300         *      4320         *      4340         *      4360         *      4380         *      4400         *      4420
Pohi(14818 : TTCCTCTTCCTTAATGATATGTGGGTTTTTGTATGAGAGTTTGCTTTTTCTCTGTTTTTTAGGTTTGTTGGTAAAGTTTGGTGTGTTTCCGTTTCAAGGTTGGATTTATAAGGTTTTATTGAGATCTGGGTGGGTTGTCGTGTGGGGATTTTCTTGTTTTTTTGAAGCTTC :  4420
          *      4440         *      4460         *      4480         *      4500         *      4520         *      4540         *      4560         *      4580         *
Pohi(14818 : CTTTTTTATTTATGTGCTTTTTTTTGTGTAGTGGTGGGAGATTCTTTTTGAGGGTATCTTGCCTTTTTTCTTTTCTTTTTTTAGGAGGGTTGTTTTGGTGTTATAGTATAGTTGATGTCATTGTTGATGCCATATGATGCTTTCTTCTAGTGCTGTGTTAGTGGCTATG :  4590
       4600         *      4620         *      4640         *      4660         *      4680         *      4700         *      4720         *      4740         *      4760
Pohi(14818 : TCTGTTAGGGGTTCTGCAGAGGCCCTTTTTTGTCTTTTTGTTATTTATGGTGTTTGAAGGAGTATGGTGATTTTATTTTCTCCCATATTGAGAAGATGGGCTTGGTGGAGGTTGGGTCATATTTTTTGTTTTGATTTTGTTGGTCTCTTTACCGATTTCTGTTTCTAT :  4760
          *      4780         *      4800         *      4820         *      4840         *      4860         *      4880         *      4900         *      4920         *
Pohi(14818 : TTTTTATAAGTTATGGATGGCGGTTAGGATTTATTTTTGTTATTTTCCGGTTTTTGTTGCTTGGTGCTTTGTATAGAATATCAGAGCAGTTGTTTTTGGTCAGGTATCTTTTAAGGATAGTGTGTCATGTGACACATATGGCGGAGTCTTTTTAGCTAGTTTTAGTTGAC :  4930
trnV   4940         *      4960         *      4980         * trnA 5000         *      5020         *      5040         *      5060         * trnD  5080         *      5100
Pohi(14818 : GAGATAGTTTAATGAAAATGTCTATTTTACACGTAGAGGTTGGTTATGGTGCCTATCGTCAGGATGACATAGTTTAAGTTAGGAGATGGCTGCTCTGCAAGTAGCCGGTGAGGGTTCCTCTGTTGTTGGGGGGGACAGCCTTAGTTTAATTGAAGGATGATAGTTGTC :  5100
          *      5120         * nad1 5140         *      5160         *      5180         *      5200         *      5220         *      5240         *      5260         *
Pohi(14818 : GTGCTATTGGTGGGTTTGATTCTCAGGCTGTGATGGTTTCTTTGTTGACGGGGGTTTATTTATTTTTAAGGAGGCTTATTTCTTTTGGTTTAATTATGCTGTTTGTGGCCTTTTTTATTTTGGGAGAGCGTAAGGTGTTAGGGTATATTCAGTTTCCTAAGGGTCCTAAT :  5270
       5280         *      5300         *      5320         *      5340         *      5360         *      5380         *      5400         *      5420         *      5440
Pohi(14818 : AAGGTAGGTTTGGTGGGATTACTACAGAGGTTTGCAGATCTTTTGAAGTTGGTTATAAAGTTTAAGGTTTCTTCCTTTCAGGGTCGTAGTTGGCTTTCTTGATGAGGGGTGGGTGTCTTGGTTCTTTTGGGTTGTCTTTATTGTGTCTTTTTCTCTTTTAGTTATTCTGG :  5440
          *      5460         *      5480         *      5500         *      5520         *      5540         *      5560         *      5580         *      5600         *
Pohi(14818 : TTTGAGGAGGAGAAATAGAGCTCTTTGGTTGTTGGTCATTACTAGTGTTACAGGGTATAGTCTTTTAAGGATAGGTTGGGGTTCTTATAAAAAGTATGCTTTATTGAGATGTTTGCGATCTGCGTTTGGTTCTGTGACTTTTGAAGCCTGTTTTATGTGTATTGTTTTAT :  5610
       5620         *      5640         *      5660         *      5680         *      5700         *      5720         *      5740         *      5760         *      5780
Pohi(14818 : TGCTGGCTATTAGTTAGGGGGATTTATTCATTTAGGTCCTTGTTTGGAGCGTTCTTTTTTATTTTATTTCTTTATCTTTTGCCCTGTTGTTATATTCTTTGATTGGTGGGTAGTTTTGTGGTGAGTGTAATCGGACTCCTTTGGACTATGCTGAGGCTGAAAGGGAGCTTGTTAGGGGG :  5780
          *      5800         *      5820         *      5840         *      5860         *      5880         *      5900         *      5920         *      5940         *
Pohi(14818 : TTGAATACGGAGTATTGTAATGTGCCGTTTACTTGCCTTTTTGCTTGTGAGTACTAATAATGTTTATTTTCTCTTGGTTAGGGAGGGTAATATTTTGAGGAGGTGGAGCTGTATTATTTTTGACTGTTTTCACGTTATTTTTTTTGTGTGGGCCCGGGCGACGTTGCC :  5950
       5960         *      5980         *      6000         *      6020         *      6040         * trnN 6060         *      6080         *      6100         *      6120
Pohi(14818 : TCGAGTTCGTTATGATTATTTTGTTCGGTTTATGTGAAAGTGGGCTGTGTTGGTTTTAGTTTTTTCTTTTTTTTTTGGTTTTGGTTTAGGGGTGAATTTGGGTCGTGTAGATTATTTTAAATCCTAAGGCTGTTAACCTTAAGAAGAGTTTTATTCGGTTCCGCGGCCAA :  6120
trnP       *      6140         *      6160         *      6180         *      6200 trnI    *      6220         *      6240         *      6260         * trnK 6280         *
Pohi(14818 : CACGGTGGTCTAAAATTTTAGGATATGAGTTTTGGGGACTCGTGGTCCCGCTAGGGCTTGTTGATTTTTTGCCGGTTGGGCTGCGTTAGCAGGTTGCTGTGATATAGCGGTTGTGAGATTTTATCTTCTGCGGTAAGCAGGTATTCTAAAAGTAGCTTATTTAAAGCTT :  6290
       6300         *      6320         * nad3 6340         *      6360         *      6380         *      6400         *      6420         *      6440         *      6460
Pohi(14818 : TGGATTCTTACTCCTAGGATGTTGCTTAAACCTTTTAGGATGGGAGTTTTGTTATGTGGTGCTGGATTAGGAGGTTTGATTCTTTTGCTGGTTTGCCTATTCCATAGTTTTGTTTGAAATATTAGGGATAGTTGGTTAGGTTTACGTTCTTGGGTAAGTTCTTTCGA :  6460
          *      6480         *      6500         *      6520         *      6540         *      6560         *      6580         *      6600         *      6620         *
Pohi(14818 : ATGTGGTTTTGTTTCTCAGCGTGTTGTTGAGGATTATTTTAGGCATACTATTTTATTTTGGTGGTTTTTTTTTGTTGTTTTGATTTGGAGATTTCTTTGTTGTTGAAAATGCCGTTGCAGGGTGTTTTGTATAAGAATTTGGGATTTTATATGTAGTTTTTCTTCTTTTGT :  6630
       6640         *      6660         *      6680         *      6700 trnS1(AGN)*      6720         *      6740         *      6760 trnW*      6780         *      6800
Pohi(14818 : TGGCGTTCGGATTTGGTGCAGAAATTATAAGGGTTATGCGGAGTGAGGTTATTAGATTGATTGGGGGCTTGTTTGGGTTGTCGCTGCTAACGAGAATTGGGGTATTAATAATATCCGGCCCCCTTTTCTGGAATAAGCTAGGTTATTGTTTGACTATCTGTTTCAAAA :  6800
          *      6820         * cox1 6840         *      6860         *      6880         *      6900         *      6920         *      6940         *      6960         *
Pohi(14818 : CAGAGGGAGGCCTTGGGCCGTTTATTGATTATGACTTGATTATTTACTTTGGATCATAAGCGTATTGGTATGATTTACATGGTCTTGGGTGTGTGAGGTGGATTTATTGGACTTTCTTTAAGAATGTTAATTCGTTTGAATTATCTTGATCCGTATTATAATATTGTCTC :  6970
       6980         *      7000         *      7020         *      7040         *      7060         *      7080         *      7100         *      7120         *      7140
Pohi(14818 : GTCGGAGGTTTACAATTATGTGATAACTAATCATGGGGTTGCTATGATTTTCTTTTTTTTGATGCCTGTGTTGATAGGAGGATTCGGTAATTATCTTCTTCCTCTTTTATTGGGTATACCTGATTTGAATCTTCCTCGTTTGAAAGCTCTAGGGCTTGATTAATGCTTC :  7140
          *      7160         *      7180         *      7200         *      7220         *      7240         *      7260         *      7280         *      7300         *
Pohi(14818 : CTTCTGCTGTTTGTCTTAGTTTGAGCATGCTAGGAGGGGCAGGAGTAGGATGAACCTTCTATCCGCCTTTATCAGGTGGGGATTATCTGGGTGAGGTACAGATTTTTTGATGTTTCTCTTCATTTGGCGGGGTTATCTAGTCTTTTGGGTTCCTTAAATTTTATTTGT :  7310
       7320         *      7340         *      7360         *      7380         *      7400         *      7420         *      7440         *      7460         *      7480
Pohi(14818 : ACTATTGTTAGGGCGTTATGCGAGCATACGGTGGGTCGTAGGTCTATAATTGTTTGGGCGTATCTTTTTACGTCTGTTTGTTACTACTTTCTTTACCAGTATTAGCGGCTGCAATTACCATGCTTTTGTTTGATCGTAAATTTAGTTCTGCTTTTTTTGATCCCTTGGG :  7480
          *      7500         *      7520         *      7540         *      7560         *      7580         *      7600         *      7620         *      7640         *
Pohi(14818 : TGGAGGTGATCCTGTTTTGTTCCAGCATTTGTTTTGGTTTTTTGGGCATCCTGAGGTTTATGTTTTAATTTTACCAGGATTTGGGATTGTGAGCATATTTGTATGACTCTAACTAATAAAGATTCGTTGTTTGGTTATTATGGATTGGTGTTTGCTATGGGGGCGATTG :  7650
       7660         *      7680         *      7700         *      7720         *      7740         *      7760         *      7780         *      7800         *      7820
Pohi(14818 : TGTGTTTGGGAAGTGTTGTCTGGGCCCACCATATGTTTATGGTTGGTTTGGATGTTAAGACTGCAGTATTTTTCAGATCTGTTACGGGGGTGATAGGTATTCCTACAGGGATTAAGGTTTTTTCTTGGTTATTTATGTTAGGGGGTACTCGTCTACGGTTTGGGATCCT :  7820
          *      7840         *      7860         *      7880         *      7900         *      7920         *      7940         *      7960         *      7980         *
Pohi(14818 : GTAATATGATGAATTCTTGGGTTTATCTTTTTGTTTTACAATAGGGGGTGTAACTGGAATTATCTTGTCTTCTTCTATTTTGGACAGTTTATTGCATGACACTTGGTTTGTTGTTGCACATTTCCATTATGTTTTACTCCTGGGATCTTATAGCACTGTTGTAATATCCT :  7990
       8000         *      8020         *      8040         *      8060         *      8080         *      8100         *      8120         *      8140         *      8160
Pohi(14818 : TCTGTGATGGTGGCCTATTGTGACCGGGTTTAGTTTAAATAAGTATTTGTTGCAAGGGCATTGGGTGGCATCAATGGTAGGGTTTAATTTTTGTTTTTTCCCCATGCATTATCTGGGGCTTATGGTCTTCCACGTCGGGTGTGTAACTATGAGCCGGCCTTTCATTGAT :  8160
          *      8180         *      8200         *      8220         *      8240         *      8260         *      8280         *      8300         *      8320         *
Pohi(14818 : TAAACAATATTTCTTCTGTTGGAGCTTTAATTTCTGTGGTTAGGGCTTTTTTTTTGGTTTTTATTCTTTGAGAGTCTCTTGTGGTTGGAAATCGTGTTGTGGGGTTTGAGGTAGCAAATCTTTGGTTATGAATGCTTTGGTTACTCCTGTGCCTCATCATAGGGTTTAC :  8330
       8340         * trnT   8360         *      8380         *      8400         *      8420         * trnL 8440         *      8460         *      8480         *      8500
Pohi(14818 : ATTAGTGGGCCGTCTCGTTGGCTTTCTTTTAGGAAGTGTAGTTTATGAGAGAATGCTGCTTTTGTAAAGCAGTGGTGTAATTTGATTAGCTTCTTTGATTTTGAGATCGTTTTAGTTTTTGTGTTTAGGGGGTGTCGTACCTTTTGCATCATGATTCTTTGACGGAAGT :  8500
          *      8520         *      8540         *      8560         *      8580         *      8600         *      8620         *      8640         *      8660         *
Pohi(14818 : TGGTTATATTCAGTGTCTCGAAAGGCAACGATTTAGGTTTATCTTGTTTTAGAACTTAGGGATTGGCGGGTAGCTGAATCTTAAAGATAGACTTGGAGGCGATAGGTAATGCGTGTTGCTTTATAGCTAGTTTTTGAGTGCTTTTTCGTTTTAGCCTTGTTGCTTAAATG :  8670
       8680         *      8700         *      8720         *      8740         *      8760         *      8780         *      8800         *      8820         *      8840
Pohi(14818 : TGATGGGGCAGTTAAGAGAGTGTATGTCTATGGTTTTAAGGTGGGTTAAAGGTACCCAGCTTTAGTTTCGTGTTATAACGATTTTTTGTCATAGGTTTTGTCTTTAGTTTGGTACTCAAATAGTTTGGAATATTTGTTTTAGTATGGAGGGATTAGTAGTTTTAATAGTTC :  8840
          *      8860         *      8880         *      8900         *      8920         *      8940         *      8960         *      8980         *      9000         *
Pohi(14818 : CTTTTTTCTCGAATTTCTTCAGTCTGTTTCTTAAAAACATTGCCATTGTTAGAATTTTGATGGTAGGGCCTGCTCGATGTTTTAATAAATGGCCGCAGTATTTTGACTGTGCTAAGGTAGCATAATTAGTTGCCTTATAATTGAAGGATTGTTTGAATGGTTTAACTAG :  9010
       9020         *      9040         *      9060         *      9080         *      9100         *      9120         *      9140         *      9160         *      9180
Pohi(14818 : AGGAGTATTTTAGATGGGGGCTATTATTGAAATTAGGTGGCTGGTGCAGATCCCAGCGTTTTTTATTAGACGGAAAGACCCCGAGAGCTTAATAATAATTTGTTGTTTACTTGGGGCAAGAGCATATGCTTTATATGTTTTTGGATCCTTTTGGAAGGAGATTAGTTAC :  9180
          *      9200         *      9220         *      9240         *      9260         *      9280         *      9300         *      9320         *      9340         *
Pohi(14818 : CTCGGGGATAACTAGGTAAGAGGCAAGGAGAGGTCATATCGATTTGCCTGTTTGCTATCTCGATGTTGACTTAGGGTGAGTATGAGGGTGCAGTAGTTTTCATACATGGTCTGTTCGACCGTAGTTCCCTTCATGAGTTGAGTTAAGACCGGCGTGAGCCAGGTCGGTTC :  9350
       9360         *      9380         *      9400 trnC *      9420         *      9440         *      9460 rrnS 9480         *      9500         *      9520
Pohi(14818 : TTATCTTTTATTGTTGCGGTTTCGTACGAAAGGAGCATCCGTGATTTAGGTTGGGCGAGTTATGTGTTTAACATAGTTTACTCTGCAAAGGTGAATTTGATTTTTTGGTCCTCGTCCTAGTTCAATTTAATTCTGGTTGTGGAAGGGGCTCAAAGTAGTACTACATAGGCT :  9520
          *      9540         *      9560         *      9580         *      9600         *      9620         *      9640         *      9660         *      9680         *
Pohi(14818 : TTGTTTCTTTCAGGAAGTTGATAGGTTTGTGGCGTTGCTTAGTGGTTGGATTTTGTTTGAGGTAACTCTGATTGGGCTGCTTTGAGGGAGGTGGAAAATTCACAGTGCCAGCATCCGCGGTTATTCTGTTAATCTCCTCTTCTAGCTGGTTTAAATGATTTGCAGGA :  9690
       9700         *      9720         *      9740         *      9760         *      9780         *      9800         *      9820         *      9840         *      9860
Pohi(14818 : GATTTCGGACTTTTGGGTAGAATTGTTGTCTTGTTGAGAAACACTTGCATTTTTTTGGAGAACTCTCTAGGAAAAGGATTAGAGACCCTTGTACTGAGTGGAGTATTTTGTGGCCATGGTGTAAACTAAAAGGATTTGGCAGCCGATGATGTTCCTCCGGGGGAGTGTGT :  9860
          *      9880         *      9900         *      9920         *      9940         *      9960         *      9980         *     10000         *     10020         *
Pohi(14818 : GGCGTAAGAGATAGTCCACTTAGGATCTTACCATCATTATTAGTTAGTGTATATCCGGTTTAAGATTCGTGATTTGTCTCATTGAGTGTTATTGATTTAATTGAATCCAGGTCAATGTGCTGCTTATGTGATGGTGCTTGTGCACTACTTTTTGAATATTCTTCAATGTA : 10030
      10040         *     10060         *     10080         *     10100         *     10120         *     10140         *     10160         *     10180         *     10200
Pohi(14818 : AATTGTGGGAGGTTTAGGACTCGGAAGTAGGCGGATTGAGGTTTGTTCTGTCGAATTTGGATTTAGTTGGGGTACATACCGCCCGTCACCCAGGGTGATAACTGTGGTAAGTCGTAACATGGTAATGTTGGGGGAACCGGACATTAGTTGGTAAATTCTTAGTTAGGAGT : 10200
          *  cox2  10220         *     10240         *     10260         *     10280         *     10300         *     10320         *     10340         *     10360         *
Pohi(14818 : ACCTATGTATGTGTATACACCATTATATTTCTGAGATGGTAGGTTATGTTTCTTTTTTGTCAGCTTTTATACCTATTTGGGTTTTTTTAGTTTTGGGATGACAGCTATGTAGATCTTATGGAACTGCAGCACTTCTGTAAAGAAAGAGATTTGTTGGAGTTTTTTTGGACGG : 10370
      10380         *     10400         *     10420         *     10440         *     10460         *     10480         *     10500         *     10520         *     10540
Pohi(14818 : TGGTTCCCAGAGTTTGTGTTGGATTTCTTTGTCTTTTAAAATTGGGTTGTTTGGGTAAAGATTTGGTTTGAAGACGCGTGGTTTGATTAAGGTGATTGGTCGACAATGATATTTGAAGTTATGACTTAAATGATGGCTGTGGGTTGTATGATTCTATGATGAGGGATTTT : 10540
          *     10560         *     10580         *     10600         *     10620         *     10640         *     10660         *     10680         *     10700         *
Pohi(14818 : ATTGATGGTGTTGATAAGCCATTGCGTGTTTATGTGGATACTCCTTATAATCTTTCGGTGACATCTAGGGACGTTATTCATTCTTTCGCTCTTCCTATTGTTAATTTGAAGGTGGATGCCATACCTGGGCGGTTAAACAAAGTCTTTTTTTGTATGAATCATGTTGGTGT : 10710
      10720         *     10740         *     10760         *     10780         *     10800 nad6    10820         *     10840         *     10860         *     10880
Pohi(14818 : TTTTGTCGGATATTGTAGGGAGTTATGTGGTGCTGGACATGCGTATATGCCTATAGTGATGGAGGTTATTGAGAAGGCCTTTGTTAAGGATTAGTGAGTATTGTATTTTTAAGGCTGTACTTTTCAAGGTTGATTAGTTTTTCTTTTATTAGGCATCCTGTAATTTATTG : 10880
          *     10900         *     10920         *     10940         *     10960         * 10980         *     11000         *     11020         *     11040         *
Pohi(14818 : TGTTCTTCTTTTGGTTAGTTCTTTAAGAATTAGTGGGTACTTGTATTCTGTTTGTGGTTGGGCTTGTCTTGGTATTTGAGTATTTTGCTTGGTTTATGTAGGGGGGATTATGTTTATTTATTTTTGTTTTTGTTTCTGTACATATCTCTAATCCGATGTCTATTGGTGGCGGAAGGG : 11050
      11060         *     11080         *     11100         *     11120         *     11140         *     11160         *     11180         *     11200         *     11220
Pohi(14818 : GCCTTGTATTTTTGATAGCTTTTGGAAGGTTTATGTTAATTTTCAGTGGGGGCCTAGGTCATAGGCTTGGAGGGTTTTGTGAGGAGAGGCACTATCTGTCGCTCTTATTTTGAGGGGTTCTCGTATTCTTTGTTTTGTTTGGTCTTAATGATTGGTTTTGTTCGGTGTTAGG : 11220
          *     11240         *     11260 trnY    *     11280         *     11300         *     11320         *     11340trnL1-CUN*     11360         *     11380         *
Pohi(14818 : GTTATTTCTGGAGAGAAGGACTCGTTTTTTCGTTAGATTACCGACTTAGTATAATAAGTGCGTGAGATTGTAAATCTTGAAGAGGGTTTTCCAGTCGGAGAGAATTCTTTTTTAAGAGTGCCAGACAAAATGGGTTTGATTTAGGATTAAATAATGGCTGCTTATGCCCT : 11390
      11400trnS2-UCN*     11420         *     11440         *     11460         *     11480trnL2-UUR*     11500         *     11520         *     11540         *trnR 11560
Pohi(14818 : CTTGCGGGCAAGGATTTTTCCGGAAGGAATTTGAAATTCTTTCTTTCATTTAGTTAATGATGCCTGCTATCAGAGTTTAAGGATGAACATGGGTGCCAGATAAAATGGGTTGGTTTTAAGCATCAAATATGAGGGTTTCTCCCCGTGTAGTTATGCGTGATCTGATATCCC : 11560
          *     11580         *     11600         *     11620 nad5    *     11640         *     11660         *     11680         *     11700         *     11720         *
Pohi(14818 : GGTGTAGTGGGATTACGTTTCGGCCGTAATTGGGGTGGTATAACATTCTATCAGAGGTGTTGGCGTTAAGGGTGGTTTTGGTTCTTGGCAGGTTGGTTTTTTGAAGAATTGGGTTAGTTGGTTTGGGGGCTTCTTTTAAAAGTTTGAAATTTATGCTTGAAAGGATGGTG : 11730
      11740         *     11760         *     11780         *     11800         *     11820         *     11840         *     11860         *     11880         *     11900
Pohi(14818 : TGGAGTTGTTGATGTTAGACGAGGTCAGTTTAATTTGTAGCTTTATGCTTTTCTGTTGTGGAAGATTAGCACTTTTGTATTGTTATCATTATTTCGCTGATTCCGGAGAAGGTGTCTTATTGTTTCCGTTAATAGTTTGGTTTTTGGGGGTGATGGGCATTTTAATTTTT : 11900
          *     11920         *     11940         *     11960         *     11980         *     12000         *     12020         *     12040         *     12060         *
Pohi(14818 : AGGAGGAGTCTTATTTTTTCTCTAGTTTATGAGAATATTTGGGGTGGTGAGGTTTCTACTAATTTTGTTTTATTCTAACAGGAGTAGAGTCCGGGCTTCCTTAATAACTTTATTCGCGTCTCGATTTGGAGATGTGTCTTTGTTTATGCTATTCTTTGATGCGGTGTC : 12070
      12080         *     12100         *     12120         *     12140         *     12160         *     12180         *     12200         *     12220         *     12240
Pohi(14818 : CTGGTGAGGGATAGTGTTGGCCTCCTTTTTTTCTTTTTTTTTCTCCTTGTTGTTTTAAGTAAGAGAGCTGGTTATCCTTTTATTTCTTGGCTTTGGAAGCTATGCGTGCTCCTACTCCTGTGAGTTCTCTTGTACATTCTTCTACTTTGGTTGCTGCAGGGGTTTGAT : 12240
          *     12260         *     12280         *     12300         *     12320         *     12340         *     12360         *     12380         *     12400         *
Pohi(14818 : TTCTTTTACGTTATCAGAGGCTTTTGGTTTCTTTTTTGAGGGGGTGATTGTGTTTGTTTTGTGTTTTGACTATTATTTTAAGGGGTATGTGTGCTTCTTTTTTTAAAGATTTGAAGAAGGTTGTTGCTTTATCGACTTGTAATAATATTTCTTGGTGTGTGGTTTTTTT : 12410
      12420         *     12440         *     12460         *     12480         *     12500         *     12520         *     12540         *     12560         *     12580
Pohi(14818 : GTCTGTGGGGATTTGTTTCTTGTTTGTTTACAGCTTTTGACGCATGGTGTTTGTAAGTGTTACTTGTTTATGTCTGTTGGTGATTTAATGTCACAGTCTGGTGGAGCAGAGTTCTGTTGGAGTATATGGTTCTCCTTATGTTTGGTGTGTTGGGTCCCATTTTGCAGAG : 12580
          *     12600         *     12620         *     12640         *     12660         *     12680         *     12700         *     12720         *     12740         *
Pohi(14818 : TGTTTTGGTTTTTTCTTTGTCTGGTCTTCCGTTTATGGGTGTTTTTTTAGGAAGCATGGTCTTTTTCGGCTGTTTCCTATTGTTATGGGTTGGGATTTTTGTTGGTGTTTTGATTTGCATTTTTTTTGACATATGTTTATTCTGTGCGTTGAGGCTACTTTTATTAA : 12750
      12760         *     12780         *     12800         *     12820         *     12840         *     12860         *     12880         *     12900         *     12920
```

```
Pohi(14818 : AGGTTTCTAGAGGTTTGTCGAGTGGTTATTTCAGTGCCTTTCTTCCTATAGCAGGTTTATGTTTGTTGGGTACGTTTTTAAATTGGTTTGGGTTGATTTTGTTTGATGAGAGTTTTAGACTGGGTATTTGGTGGGGTAGTATGATGTTGTTGGTTCAAGCTTTAGGGTGT : 12920
                     *     12940         *     12960         *     12980         *     13000         *     13020         *     13040         *     13060         *     13080         *
Pohi(14818 : TTGATGGGGTGGTTTTTGTTTTTGGGTGTGTTTAGATCTGGTGTTTGGGTTGTTTGAAATTCTTTATTATGGGGTAGGGATTGTTTGGTAGGGTTTATTTATGATGGGTTTTTGAAGTTAACAGAGGTAGCGGTTCTTTCTTTTTATCGGTGGGAGCTTTATAGTTTGGC : 13090
                 13100         *     13120         *     13140         *     13160         *     13180         *     13200         *     13220 trnG    *     13240         *     13260
Pohi(14818 : ACTTTTTAAGGCTGGTGGTGATAATTCATTTATAGGAGTTTTTACCTCTTTGAAATTATTGGTGTTGGGTAGCGTTTTTGTGATTTTTTTGTTTTCTCTCTTTTTTTGTTAAGTGGTGGTTTATGCTAGTAGTATATTTTGAGTGAAGTATTCTGTCTTTCCAAGTCAGA : 13260
                     *     13280         *     13300         *  !> 13320LR1      *     13340         *     13360         *     13380         *     13400         *     13420         *
Pohi(14818 : GGGCCATGTGTTTTGGCTAGCATAGTATTATGATTTCTAAGTCTGTTTGGGTTTAGAGGGAGTTTAAAAAATTATCCGATGTTCGTTGTTAAATAATTCGTAAGGGGGGGGTATTGTGAAAAAAAAATTTTTTGTAAATTTGTAGCTTAGTACAGGTATTCTTGAAGAGA : 13430
                 13440         *     13460         *     13480         *     13500         *     13520         *     13540         *     13560         *     13580         *     13600
Pohi(14818 : GGATGGCCTGTGGTGTTCCCCTTAATTTAGCTTTTATGTTAAAAAAATTTTTACTTTTGATTTAAAAATTTTATGTGGCTTTTTTCTCGGTAGTTTTGTGGGACAAATTTTGGTTTTTTGTGGAACTGGAATTTGATTTACTAGTATGTATATCATACAGTGAGTTATGA : 13600
               <!    *     13620trnE     *     13640         *  !> 13660LR2      *     13680         *     13700         *     13720         *     13740         *     13760         *
Pohi(14818 : TGTGGATTGTTTGTCGGTTAGGTGTAGAAGGGTTGCACGTTAATTTTTCGTGTTAGAGGGAGTTTAAAAAATTATCCGATGTTCGTTGTTAAATAATTCGTAAGGGGGGGGTATTGTGAAAAAAAAATTTTTTGTAAATTTGTAGCTTAGTACAGGTATTCTTGAAGAGA : 13770
                 13780         *     13800         *     13820         *     13840         *     13860         *     13880         *     13900         *     13920         *     13940
Pohi(14818 : GGATGGCCTGTGGTGTTCCCCTTAATTTAGCTTTTATGTTAAAAAAATTTTTACTTTTGATTTAAAAATTTTATGTGGCTTTTTTCTCGGTAGTTTTGTGGGACAAATTTTGGTTTTTTGTGGAACTGGAATTTGATTTACTAGTATGTATATCATACAGTGAGTTATGA : 13940
              <!     *     13960STR1     *     13980         *     14000         *     14020         *     14040         *     14060         *     14080STR2     *     14100         *
Pohi(14818 : TgTGCTTTCCTTCCAATCTGAGGTTGTTGAGTAATAATATTAGAAGGGGATAAAAGGTAGATGTAAAGTAGATTGGTTGTTAGGATATGAAATCCTCTAGCCGAGCCCGGTGTTGTTTGTTGGGCGAGGGGAGAGAGAGGTTGTTGAGTAATAATATTAGAAGGGGATAA : 14110
                 14120         *     14140         *     14160         *     14180         *     14200STR3     *     14220         *     14240         *     14260         *     14280
Pohi(14818 : AAGGTAGATGTAAAGTAGATTGGTTGTTAGGATATGAAATCCTCTAGCCGAGCCCGGTGTTGTTTGTTGGGCGAGGGGAGAGAGAGGTTGTTGAGTAATAATATTAGAAGGGGATAAAAGGTAGATGTAAAGTAGATTGGTTGTTAGGATATGAAATCCTCTAGCCGAGC : 14280
                     *     14300        *STR4 14320         *     14340         *     14360         *     14380         *     14400         *     14420         * STR5 14440         *
Pohi(14818 : CCGGTGTTGTTTGTTGGGCGAGGGGAGAGAGAGGTTGTTGAGTAATAATATTAGAAGGGGATAAAAGGTAGATGTAAAGTAGATTGGTTGTTAGGATATGAAATCCTCTAGCCGAGCCCGGTGTTGTTTGTTGGGCGAGGGGAGAGAGAGGTTGTTGAGTAATAATATTA : 14450
                 14460         *     14480         *     14500         *     14520         *     14540    STR6 *     14560         *     14580         *     14600         *     14620
Pohi(14818 : GAAGGGGATAAAAGGTAGATGTAAAGTAGATTGGTTGTTAGGATATGAAATCCTCTAGCCGAGCCCGGTGTTGTTTGTTGGGCGAGGGGAGAGAGAGGTTGTTGAGTAATAATATTAGAAGGGGATAAAAGGTAGATGTAAAGTAGATTGGTTGTTAGGATATGAAATCC : 14620
                     *     14640         *     14660         *     14680         *     14700         *     14720         *     14740         *     14760         *     14780         *
Pohi(14818 : TCTAGCCGAGCCCGGTGTTGTTTGTTGGGCGAGGGGAGAGAGAGGTTGTTGAGTAATAATATTAGAAGGGGATAATCATTTGGGGGGACTATAGGGGGGCAGACCTATCCATATCTTATCTTCTAAAATATTTTGGGGATTTATCTCGGCATTATTTATTGTGAGCGTTT : 14790
                 14800         *   14818
Pohi(14818 : TGTGAGCGTTTTTTTTGTGGGCTGAGAA
```
